# An empirical evaluation of sampling methods for the classification of imbalanced data

**DOI:** 10.1371/journal.pone.0271260

**Published:** 2022-07-28

**Authors:** Misuk Kim, Kyu-Baek Hwang

**Affiliations:** Department of Computer Science and Engineering, Graduate School, Soongsil University, Seoul, Korea; University of Pennsylvania, UNITED STATES

## Abstract

In numerous classification problems, class distribution is not balanced. For example, positive examples are rare in the fields of disease diagnosis and credit card fraud detection. General machine learning methods are known to be suboptimal for such imbalanced classification. One popular solution is to balance training data by oversampling the underrepresented (or undersampling the overrepresented) classes before applying machine learning algorithms. However, despite its popularity, the effectiveness of sampling has not been rigorously and comprehensively evaluated. This study assessed combinations of seven sampling methods and eight machine learning classifiers (56 varieties in total) using 31 datasets with varying degrees of imbalance. We used the areas under the precision-recall curve (AUPRC) and receiver operating characteristics curve (AUROC) as the performance measures. The AUPRC is known to be more informative for imbalanced classification than the AUROC. We observed that sampling significantly changed the performance of the classifier (paired *t*-tests *P* < 0.05) only for few cases (12.2% in AUPRC and 10.0% in AUROC). Surprisingly, sampling was more likely to reduce rather than improve the classification performance. Moreover, the adverse effects of sampling were more pronounced in AUPRC than in AUROC. Among the sampling methods, undersampling performed worse than others. Also, sampling was more effective for improving linear classifiers. Most importantly, we did not need sampling to obtain the optimal classifier for most of the 31 datasets. In addition, we found two interesting examples in which sampling significantly reduced AUPRC while significantly improving AUROC (paired *t*-tests *P* < 0.05). In conclusion, the applicability of sampling is limited because it could be ineffective or even harmful. Furthermore, the choice of the performance measure is crucial for decision making. Our results provide valuable insights into the effect and characteristics of sampling for imbalanced classification.

## Introduction

In classification problems, an imbalanced dataset is a dataset in which the number of data examples of some classes is much smaller than that of other classes. Imbalanced datasets are common in many fields such as chemical and biomedical engineering, financial management, and information technology [1]. Typical imbalanced classification problems include fraud detection [2], fault diagnosis [3, 4], anomaly detection [5, 6], disease diagnosis [7], e-mail foldering [8], face recognition [9], and oil spill detection [10]. Most machine learning methods assume equal misclassification costs between the majority and minority class examples. Therefore, they are based on the assumption that the class distribution is balanced. Thus, general machine learning algorithms show suboptimal performance on imbalanced datasets, resulting in a classifier that is biased toward the majority class [10–14].

There are two categories of imbalanced data: intrinsic and extrinsic [15]. Intrinsic imbalanced data are generated because of the nature of problem domains. Examples of such domains include credit card fraud detection [2] and disease diagnosis [14]. In contrast, an extrinsic imbalance is caused by other factors such as biased sampling processes. For example, even if data are obtained from a balanced continuous stream, data transfer could be interrupted for a specific period, resulting in an imbalanced dataset [15]. We focused on the classification of intrinsic imbalanced data where the true class distribution is imbalanced.

Three main approaches can be employed to tackle the imbalanced classification problem. The first is the algorithm level approach, which modifies or creates machine learning algorithms for imbalanced classification [16–22]. It requires an in-depth knowledge of both algorithms and application domains [14, 23]. Second, the data level approach balances the imbalanced class distribution by sampling before applying machine learning algorithms. Examples of data level approaches include oversampling to increase data in the minority classes and undersampling to reduce data in the majority classes [13, 24, 25]. Finally, the cost-sensitive learning-based approach addresses the imbalanced classification problem by assigning different misclassification costs to each class [26, 27]. In this approach, the most important and challenging process is defining the misclassification costs of different classes whose true values are unknown [28].

This work focused on the data level approach, which is the easiest and most popular of the three. This approach is easy to use because machine learning algorithms need not be created or modified [23]. Furthermore, it can be used with any machine learning algorithm. Because of its simplicity and applicability, data sampling has been more widely employed than the other two approaches [1, 14, 23]. Despite its popularity however, its effectiveness has not been rigorously validated. Rigorous validations are required because sampling inevitably distorts the class distribution of training data, increasing the discrepancy between the training data balanced by sampling and inherently imbalanced test data. This increased discrepancy could reduce the test performance of a learned classifier. In addition, undersampling could increase the variance of a learned model due to the reduced training data, resulting in reduced test performance. Therefore, a tradeoff exists between these adverse effects of sampling and their positive effect in reducing bias toward the majority class.

Numerous studies have suggested that data balancing by sampling helps improve the classifiers’ performance [12, 24, 25, 29–38]. More than half of these studies [24, 25, 29–33] mainly used decision tree classifiers such as C4.5 and C5.0. Some studies used the naïve Bayes classifier [25] and the *k* nearest neighbor method [25, 33]. Other studies evaluated the sampling method using linear discriminant analysis (LDA) [35, 36], random forests (RFs) [37], and convolutional neural networks [34]. Only a few studies have compared several classifiers [12, 38]. Japkowicz and Stephen compared C5.0, multilayer perceptrons (MLPs), and support vector machines (SVMs), showing that MLPs and SVMs were less affected by sampling than C5.0 [12]. Khushi et al. compared SVMs, logistic regression, and RFs to analyze the effect of sampling for imbalanced classification [38]. They recommended oversampling combined with RFs for the prediction of lung cancer incidence.

One limitation of previous studies is that they did not comprehensively analyze the effect of sampling using a wide range of popular classifiers including regularized logistic regression and boosting. Another limitation is that most previous studies used two famous performance measures for classification: accuracy and area under the receiver operating characteristics curve (AUROC) [1, 12, 24, 25, 29–31, 34–38]. However, several studies [39–41] suggested that the area under the precision-recall curve (AUPRC) is more informative for evaluating the results of imbalanced classification. To the best of our knowledge, no study has validated the effectiveness of sampling using AUPRC.

Unlike previous studies, our work comprehensively analyzed the effect of seven widely-used sampling methods combined with eight machine learning methods—56 combinations in total—on imbalanced classification using AUPRC and AUROC as the performance indicators. We empirically analyzed the impact of sampling using 31 real-world imbalanced datasets. In this work, we sought to answer the following questions. First, to what extent is the sampling method effective? More precisely, what is the chance of a sampling method to improve the performance of a classifier? Second, which sampling method is more effective than others? Third, which machine learning classifier is likely to be enhanced by sampling? Fourth, do we need sampling to obtain the optimal classifier for imbalanced classification? Finally, what is the effect of performance measures on evaluation of the sampling method? We present our empirical findings related to the questions above and other aspects of imbalanced classification, providing insights on using sampling to tackle the imbalanced classification problem.

## Materials and methods

### Imbalanced datasets

We used 31 binary classification datasets whose imbalance ratios, i.e., the majority class size divided by the minority class size, ranged from 1.14 to 577.88 [42–44] (see S1 Table for more details). Among the 31 datasets, 29 were obtained from the UC Irvine Machine Learning Repository (https://archive.ics.uci.edu). Among these 29 binary classification datasets, seven were originally binary and the other 22 were obtained from 14 multiclass datasets from the UC Irvine Machine Learning Repository. To develop a binary classification dataset, we grouped a set of classes of a multiclass dataset and designated them as the minority class. The remaining classes of the multiclass dataset were grouped and set as the majority class. According to the grouping procedure, more than one binary classification dataset was prepared from a multiclass dataset (see S2 Table for more details). This approach of converting multiclass datasets to imbalanced binary classification datasets has been widely employed in previous studies to increase the number of datasets used in the experiment [23–25, 32, 35]. The 30th (Creditcard in S1 Table) and 31st (Fraud_Detection in S1 Table) datasets were credit card fraud detection datasets from Kaggle (https://www.kaggle.com), which were originally binary. We downloaded all datasets on September 13, 2020, except for the Fraud_Detection dataset, which was downloaded on March 11, 2022. The access URLs for the 31 datasets are shown in S3 Table. Among the 31 datasets, nine were originally binary, and the remaining 22 were made from multiclass datasets (see S2 Table).

The Fraud_Detection dataset had 395 features. Among them, we removed two features, for which more than 90% of the examples contained missing values. Missing values of the remaining features were imputed by the median if the feature was numerical. Missing values of a categorical feature was denoted by a new category called “missing.” Then, categorical features of the Fraud_Detection dataset were encoded by one-hot encoding if the number of categories was less than five, or by weight of evidence encoding otherwise. For one-hot encoding, we used OneHotEncoder of the scikit-learn package of Python (version 1.1.1). For weight of evidence encoding, we used WOEEncoder of the category_encoders Python package (version 0.1.4). Through the encoding process, the number of features of the Fraud_Detection dataset increased to 410 (see S1 Table). The 410 features were normalized by removing the mean and scaling to unit variance using StandardScaler of the scikit-learn package of Python (version 1.1.1). We calculated each feature’s mean and variance values using the training dataset. The detailed experimental procedure including the division of the dataset into training and test sets is described in the Performance evaluation and comparison section. All datasets, except for Fraud_Detection, were processed as follows. Categorical features were represented using dummy variables. We used RobustScaler of the scikit-learn package of Python (version 0.24.1) to normalize the features. All features, including dummy variables, were normalized by subtracting the median and dividing by the interquartile range (IQR). We calculated each feature’s median and IQR values using the training dataset.

### Sampling methods for imbalanced classification

We evaluated three oversampling methods: random oversampling, synthetic minority oversampling technique (SMOTE) [25], and borderline SMOTE [32]. Three undersampling methods, i.e., random undersampling, condensed nearest neighbors undersampling [45], NearMiss2 [33], were also examined. In addition, we tested SMOTETomek [24], which is a hybrid method that combines oversampling and undersampling techniques.

In random oversampling, a set of randomly selected examples of the minority class are duplicated to increase the size of the minority class. SMOTE [25] synthesizes a new instance of the minority class using a randomly selected example of the minority class and its *k* nearest neighbors of the same class. Borderline SMOTE [32] is a variant of SMOTE that increases the number of examples of the minority class along the borderline in the feature space to focus on the “difficult-to-classify” region. Random undersampling randomly removes examples of the majority class. Condensed nearest neighbors undersampling [45] excludes the majority class example from training if its nearest neighbor is from the same class. NearMiss2 [25, 33] selects examples of the majority class with the shortest average distance from the three farthest examples of the minority class. SMOTETomek [24] is a hybrid method in which SMOTE is applied first and then, an undersampling method (the Tomek links method) is performed. The Tomek links method removes a pair of the majority and minority class examples if they are the nearest neighbors of each other. A detailed description of the seven sampling methods is provided in S1 File.

All sampling methods, except for condensed nearest neighbors undersampling, were used to balance the class distribution, making the majority to minority classes ratio one to one. The resulting class distribution by condensed nearest neighbors undersampling varies depending on the composition of a given dataset. We show the class distribution of the 31 datasets balanced by condensed nearest neighbors undersampling in S4 Table.

For conciseness and clarity, we denote random oversampling by O_Random, SMOTE by O_SMOTE, borderline SMOTE by O_Border, random undersampling by U_Random, condensed nearest neighbors undersampling by U_Condensed, and NearMiss2 by U_NearMiss. We used the implementation of the seven sampling methods from version 0.7.0 of the imbalanced-learn package of Python (https://imbalanced-learn.org) [46].

### Machine learning methods

We evaluated the seven sampling methods using eight machine learning methods, which included four non-linear methods—adaptive boosting (AdaBoost), extreme gradient boosting (XGBoost), RFs, and SVMs—and four linear methods—the LDA and three regularized logistic regression methods, i.e., ridge, lasso, and elastic net. For XGBoost, we used the XGBoost Python package (version 1.4.2) (https://xgboost.readthedocs.io). For the other machine learning methods, we used scikit-learn package of Python version 0.24.1 (https://scikit-learn.org) [47]. We adopted the radial basis function kernel for SVMs. The hyperparameters of each machine learning method were optimized using cross-validation (CV) on the training dataset. We used the whole training dataset to optimize the hyperparameters except when optimizing the hyperparameters of SVMs for the largest dataset, i.e., Fraud_Detection (590,540 examples x 410 features) (see S1 Table). This dataset was more than seven times larger than the second largest dataset, i.e., Covtype4 (581,012 examples x 54 features). The training time for SVMs is affected more by the size of the training dataset than the other machine learning methods. Therefore, 10% of the training dataset of Fraud_Detection was randomly selected and used for hyperparameter optimization of SVMs. The hyperparameter optimization process is detailed in the next subsection.

### Performance evaluation and comparison

**Fig 1:**
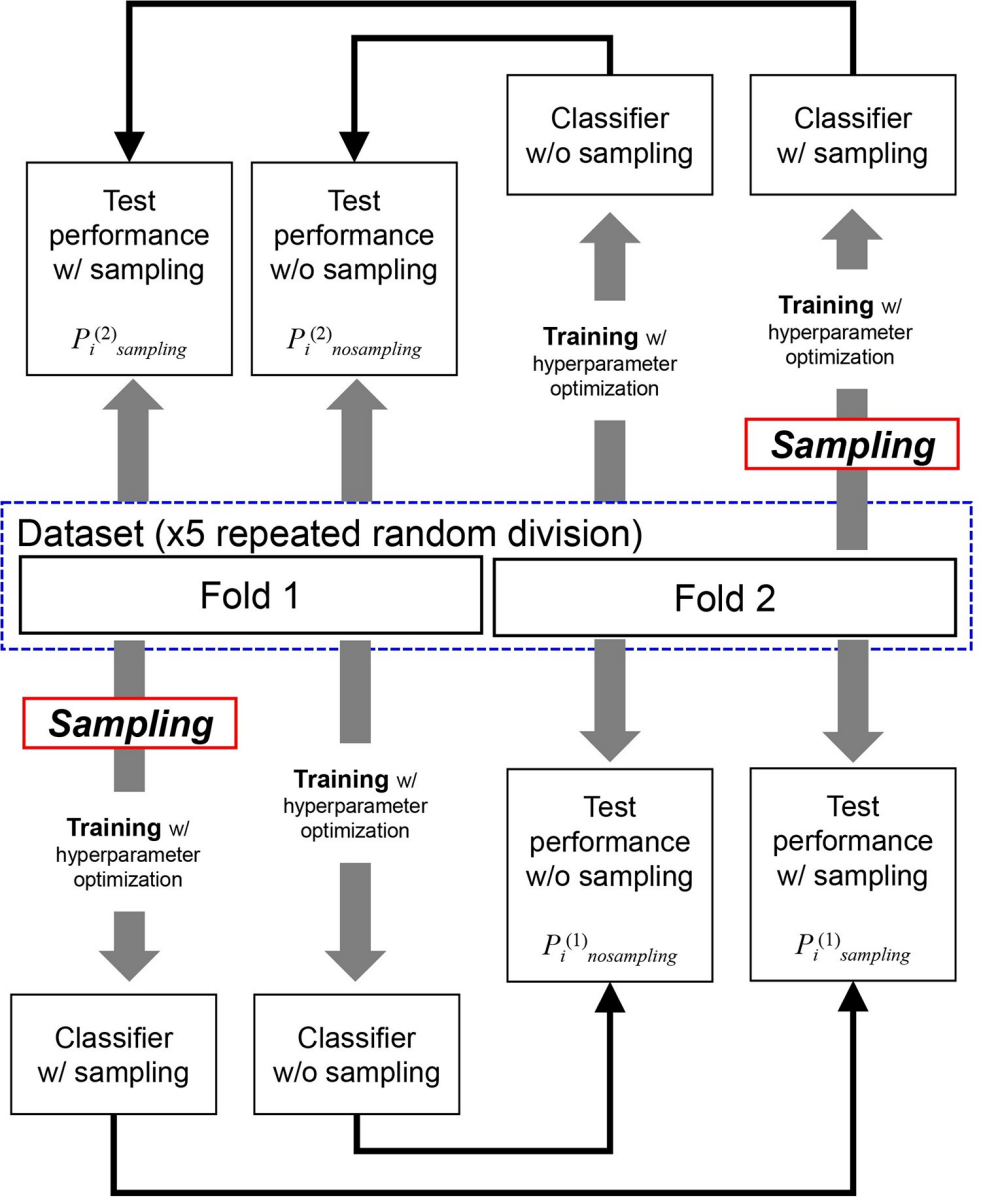
Schematic diagram of the workflow for evaluating the effectiveness of sampling for imbalanced classification. The process is repeated five times (*i* = 1, 2, 3, 4, 5) with repeated random division of an imbalanced classification dataset.

We used both AUPRC [48] and AUROC [49] to evaluate the classification performance. These performance measures were calculated using version 0.24.1 of the scikit-learn package of Python (https://scikit-learn.org) [47]. To compare the performances of the two classifiers on an imbalanced classification dataset, we performed the 5x2 CV paired *t*-test, as described in [50], in which a stratified two-fold CV is repeated five times. The 5x2 CV procedure for evaluating the effectiveness of the sampling methods is as follows, and a schematic diagram is shown in Fig 1.

First, an imbalanced classification dataset was randomly divided into two folds: fold 1 and fold 2. The class ratio in each fold was similar to that of the original imbalanced dataset. Fold 1 was used as the training dataset. A sampling method was applied to fold 1, thereby balancing the class ratio. Then, a machine learning classifier was learned from the balanced training dataset, i.e., balanced fold 1. When training the classifier, we optimized its hyperparameters using 3-fold CV on balanced fold 1. A grid search over a range of hyperparameter values was performed using sklearn.model_selection.GridSearchCV from the scikit-learn package of Python (version 1.1.1). A list of the optimized hyperparameters of each machine learning method and their search ranges are described in S5 Table. The same performance measure was used for evaluating the classifier—AUPRC or AUROC—when optimizing the hyperparameters. The classifier learned with sampling was evaluated using fold 2 as the test dataset. We denote the test performance with sampling by *P*_1_^(1)^_*sampling*_. For comparison, a classifier without sampling was learned using the original fold 1 as the training dataset. The hyperparameters of the classifier were optimized on fold 1. The learned classifier without sampling was evaluated using fold 2. We denote the test performance without sampling by *P*_1_^(1)^_*nosampling*_. Then, the first performance difference value due to sampling was calculated as *P*_1_^(1)^ = *P*_1_^(1)^_*nosampling*_−*P*_1_^(1)^_*sampling*_. The above procedure was repeated using fold 2 as the training dataset and fold 1 as the test dataset, resulting in the second performance difference value due to sampling: *P*_1_^(2)^ = *P*_1_^(2)^_*nosampling*_−*P*_1_^(2)^_*sampling*_. The procedure for obtaining two performance difference values was iterated five times, with repeated random division of the imbalanced dataset, producing five pairs of performance difference values: (*P*_1_^(1)^, *P*_1_^(2)^), (*P*_2_^(1)^, *P*_2_^(2)^), (*P*_3_^(1)^, *P*_3_^(2)^), (*P*_4_^(1)^, *P*_4_^(2)^), and (*P*_5_^(1)^, *P*_5_^(2)^). Then, the *t*-statistic for the test was calculated as follows [50]:

$$t=\frac{P_{1}^{\left(1\right)}}{\sqrt{\frac{1}{5}\sum_{i=1}^{5}s_{i}^{2}}}.$$ (1)

In Eq (1), *s*_*i*_^2^ denotes the estimated variance of the *i*th iteration (*i* = 1, 2, 3, 4, 5), calculated as follows.

$$s_{i}^{2}={\left(P_{i}^{\left(1\right)}-\bar{P_{i}}\right)}^{2}+{\left(P_{i}^{\left(2\right)}-\bar{P_{i}}\right)}^{2},$$ (2)

where $\bar{P_{i}}=(P_{i}^{\left(1\right)}+P_{i}^{\left(2\right)})/2$. To evaluate the effectiveness of sampling for imbalanced classification, we performed a two-tailed test using the *t*-statistic.

## Results

### Effectiveness of sampling methods

**Fig 2:**
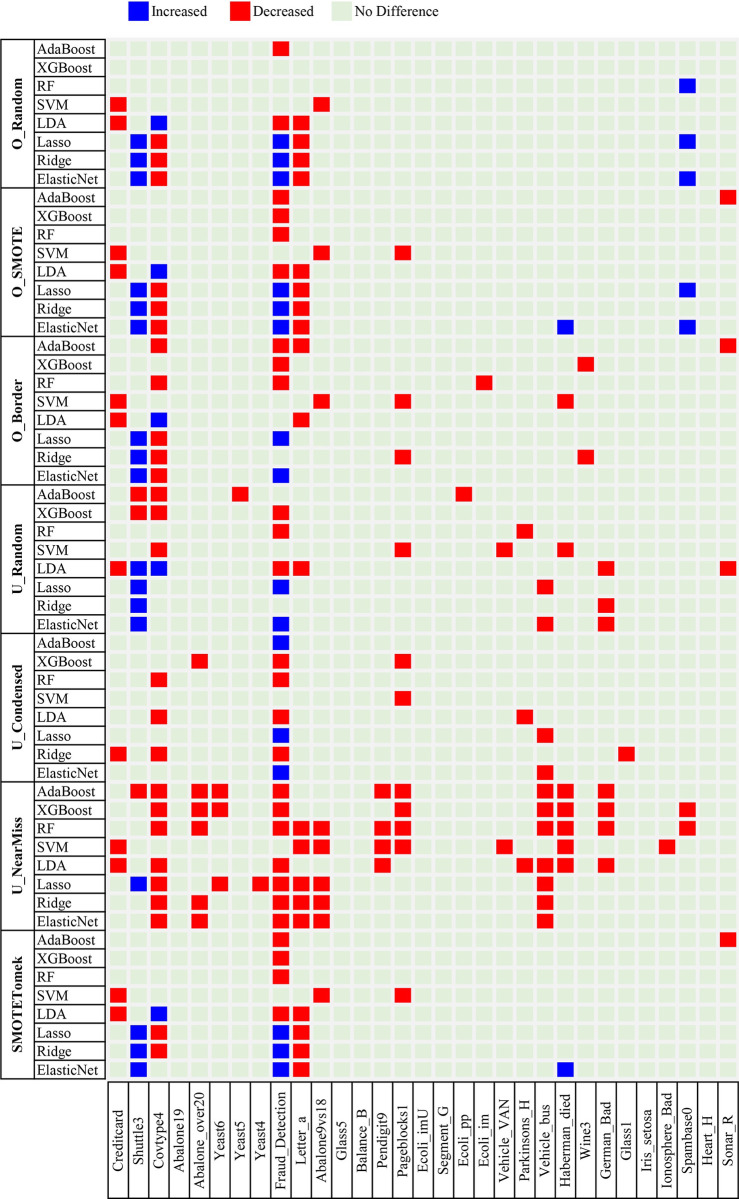
Heatmap of the difference in the area under the precision-recall curve between classification with and without sampling on the 31 imbalanced datasets. Combinations of the seven sampling methods [i.e., random oversampling (O_Random), synthetic minority oversampling technique (O_SMOTE), borderline synthetic minority oversampling technique (O_Border), random undersampling (U_Random), condensed nearest neighbors undersampling (U_Condensed), NearMiss2 (U_NearMiss), and SMOTETomek] and eight machine learning methods [i.e., adaptive boosting (AdaBoost), extreme gradient boosting (XGBoost), random forests (RFs), support vector machines (SVMs), the linear discriminant analysis (LDA), lasso, ridge, and elastic net] were compared using the 31 datasets.

To investigate the effectiveness of the sampling methods for imbalanced classification, we checked whether sampling changed the classification performance of a machine learning algorithm. The hyperparameters of each machine learning algorithm (see S5 Table) were optimized as described in the Materials and Methods section, and the optimized hyperparameter values are provided as S1 and S2 Datasets. The comparison results for each combination of machine learning and sampling methods on the 31 datasets are shown in Figs 2 (for AUPRC) and 3 (for AUROC).

**Fig 3:**
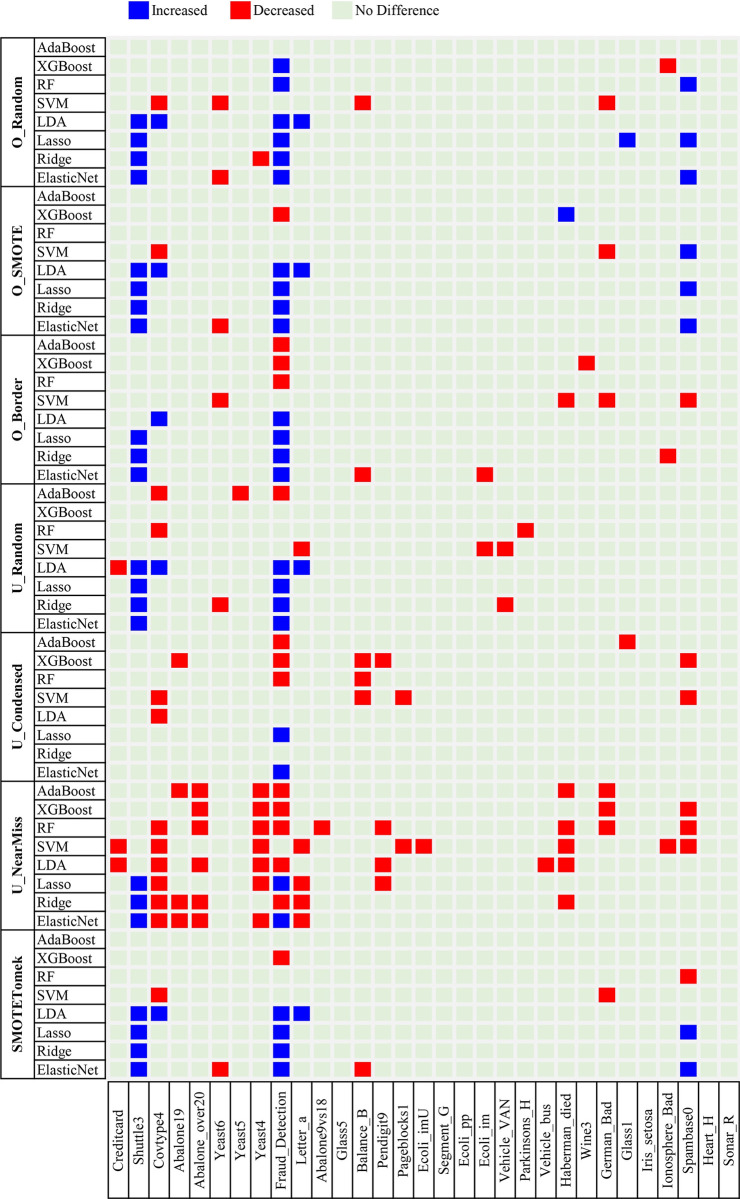
Heatmap of the difference in the area under the receiver operating characteristics curve between classification with and without sampling on the 31 imbalanced datasets. Combinations of the seven sampling methods [i.e., random oversampling (O_Random), synthetic minority oversampling technique (O_SMOTE), borderline synthetic minority oversampling technique (O_Border), random undersampling (U_Random), condensed nearest neighbors undersampling (U_Condensed), NearMiss2 (U_NearMiss), and SMOTETomek] and eight machine learning methods [i.e., adaptive boosting (AdaBoost), extreme gradient boosting (XGBoost), random forests (RFs), support vector machines (SVMs), the linear discriminant analysis (LDA), lasso, ridge, and elastic net] were compared using the 31 datasets.

Figs 2 and 3 show that there are few cases of statistically significant performance changes due to sampling. Among the 1736 (combinations of seven sampling and eight machine learning methods, applied to 31 datasets) cases, only 211 (12.2%) and 173 (10.0%) showed statistically significant differences (paired *t*-tests *P* < 0.05) in the AUPRC and AUROC, respectively. Surprisingly, we observed more cases of performance degradation than improvement, indicating that sampling could be more harmful than beneficial for imbalanced classification. Among the cases of performance changes, the proportion of cases with decreased AUPRC and AUROC was 78.7% (166 of 211) and 61.3% (106 of 173), respectively. These observations also suggest that sampling is more effective when measuring the performance using AUROC than when using AUPRC. While more cases with decreased AUPRC (166 cases) were observed than those with decreased AUROC (106 cases), the number of cases with increased AUROC (67 cases) was greater than that with increased AUPRC (45 cases).

We divided the 31 imbalanced datasets used in the experiment into two categories: nine originally binary datasets and 22 binary datasets made from multiclass datasets by merging two or more classes (see Materials and Methods and S2 Table). The numbers of cases in the nine originally binary datasets and the other 22 datasets were 504 (combinations of seven sampling and eight machine learning methods, applied to nine datasets) and 1232 (combinations of seven sampling and eight machine learning methods, applied to 22 datasets), respectively. Interestingly, sampling effectiveness differed by the category of datasets. The proportion of cases showing statistically significant performance changes due to sampling was much higher for the originally binary datasets [17.3% (87 of 504) in AUPRC and 15.3% (77 of 504) in AUROC] than for the other datasets [10.1% (124 of 1232) in AUPRC and 7.8% (96 of 1232) in AUROC]. However, both dataset categories showed a similar pattern, i.e., sampling was more likely to reduce than improve the classification performance. In AUPRC, 73.6% (64 of 87) of the cases for the originally binary datasets and 82.3% (102 of 124) of the cases for the other datasets exhibited performance degradation. The AUROC was reduced in 54.5% (42 of 77) of the cases for the originally binary datasets and 66.7% (64 of 96) of the cases for the other datasets. Furthermore, the effect of performance measures was the same for both categories of datasets. The number of cases with increased AUROC (35 cases for the originally binary datasets and 32 cases for the other datasets) was greater than that with increased AUPRC (23 cases for the originally binary datasets and 22 cases for the other datasets), whereas more cases with decreased AUPRC (64 cases for the originally binary datasets and 102 cases for the other datasets) were observed than those with decreased AUROC (42 cases for the originally binary datasets and 64 cases for the other datasets).

We examined the relationship between the effectiveness of the sampling methods and the degree of imbalance of the data. S1 Fig shows the scatterplots of the number of cases of performance reduction or improvement against the ratio (in a logarithmic scale) of the majority to minority classes for the 31 datasets. To each dataset, 56 combinations of sampling and machine learning methods were applied (see Materials and Methods). We observed small but statistically significant positive correlations between the logarithmic imbalance ratio and the number of cases of performance improvement or degradation due to sampling (the Pearson correlation coefficient tests *P* < 0.05). The R^2^ value was 0.13 for an increase in AUPRC, 0.14 for a decrease in AUPRC, 0.11 for an increase in AUROC, and 0.11 for a decrease in AUROC. The positive correlation is attributed to the fact that the amount of training data modification caused by sampling is proportional to the imbalance ratio.

### Effectiveness comparison of sampling methods

**Fig 4:**
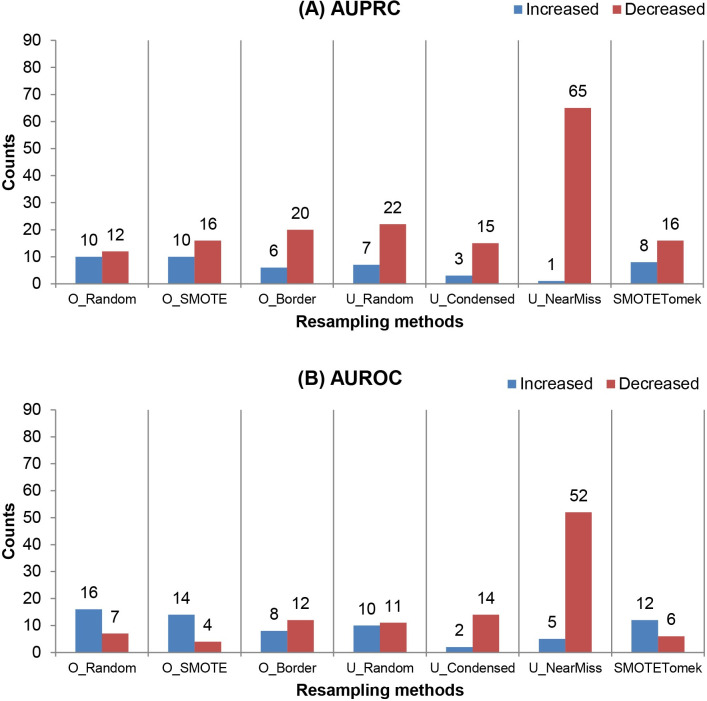
Comparison of the effectiveness of the seven sampling methods. The number of cases in which a sampling method enhanced (blue) or reduced (red) the performance in (A) the area under the precision-recall curve (AUPRC) and (B) the area under the receiver operating characteristics curve (AUROC) is shown. Seven sampling methods—random oversampling (O_Random), synthetic minority oversampling technique (O_SMOTE), borderline synthetic minority oversampling technique (O_Border), random undersampling (U_Random), condensed nearest neighbors undersampling (U_Condensed), NearMiss2 (U_NearMiss), and SMOTETomek—were compared.

We evaluated and compared the effectiveness of seven sampling methods using the number of cases in which each sampling method enhanced or reduced the classification performance. The total number of cases for each sampling method was 248 (eight machine learning methods and 31 datasets). The results of the comparison are shown in Fig 4.

The best method to improve the AUPRC and AUROC was O_Random. O_SMOTE was also the best for increasing the AUPRC. U_NearMiss and U_Condensed were the least effective in improving the classification performance in terms of AUPRC and AUROC, respectively. Notably, U_NearMiss decreased both AUPRC and AUROC in much larger numbers of cases compared with the others. U_NearMiss was also observed to perform poorly in a previous study [38]. On average, undersampling reduced the classification performance in more cases than oversampling and hybrid methods. One explanation for the relatively stronger negative effect of undersampling is that some characteristics of the data that are helpful for discriminating the majority class are removed during the process of eliminating examples of the majority class [16]. Moreover, the application of undersampling could increase the model variance by reducing the training dataset size. Furthermore, we found that AUPRC was more likely to be reduced by sampling than AUROC regardless of the sampling methods applied.

### Comparison of machine learning methods by the effectiveness of sampling

**Fig 5:**
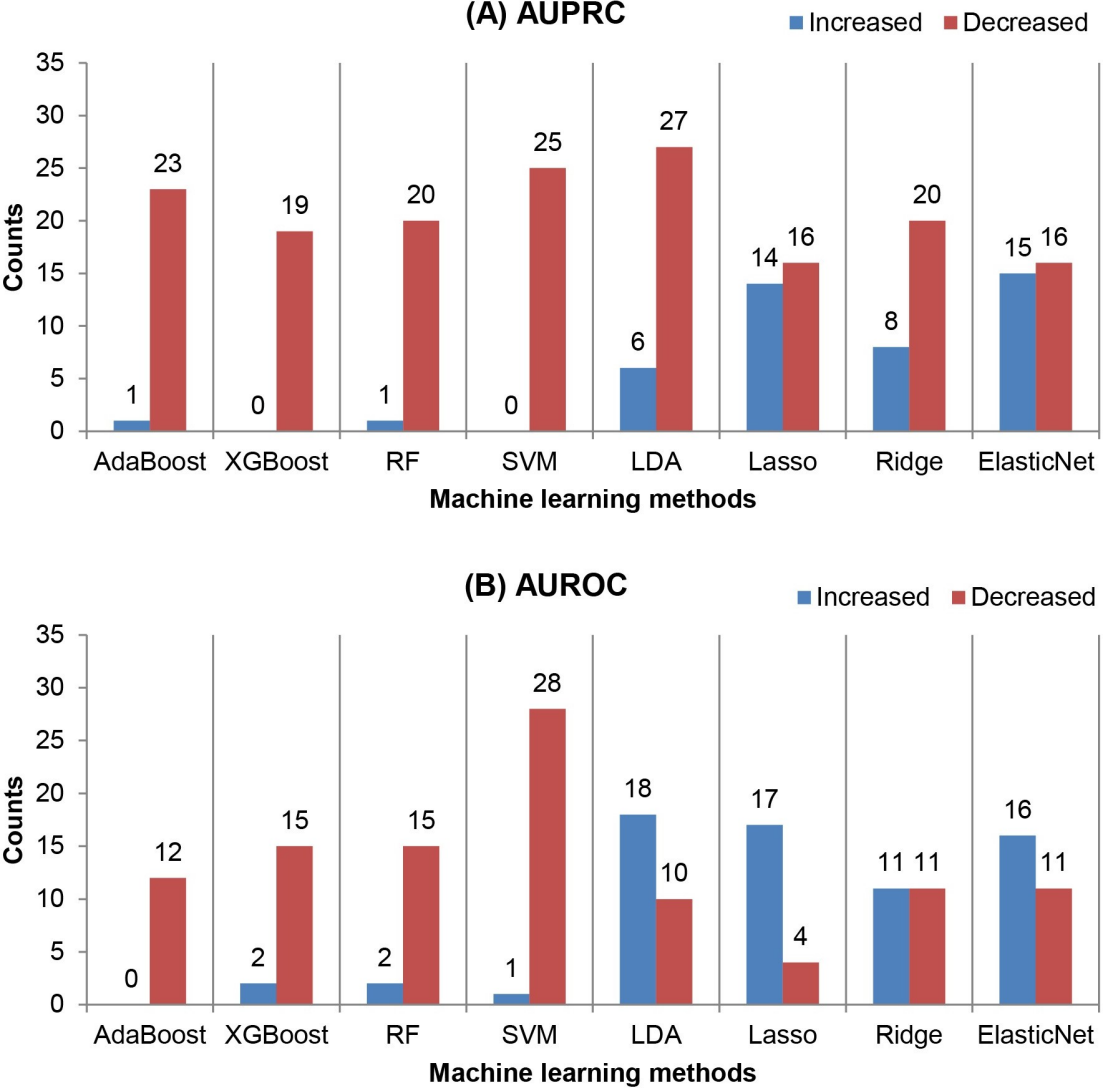
Comparison of machine learning methods by the effectiveness of sampling. The number of cases in which (A) the area under the precision-recall curve (AUPRC) and (B) the area under the receiver operating characteristics curve (AUROC) of a machine learning method were improved (blue) or reduced (red) by sampling. Eight machine learning methods—adaptive boosting (AdaBoost), extreme gradient boosting (XGBoost), random forests (RFs), support vector machines (SVMs), the linear discriminant analysis (LDA), lasso, ridge, and elastic net—were compared.

Next, we investigated whether machine learning model selection made a difference in the effectiveness of sampling. We compared the number of cases in which sampling improved or reduced the performance of each machine learning classifier. A total of 217 cases were studied for each classifier (seven sampling methods and 31 datasets). Fig 5 shows the comparison results.

We found that sampling was much more effective in improving the linear machine learning methods (i.e., the LDA, lasso, ridge, and elastic net) than the non-linear methods (i.e., AdaBoost, XGBoost, RFs, and SVMs). The elastic net and LDA classifiers showed the largest number of AUPRC and AUROC enhancement cases, respectively. In contrast, sampling did not enhance the AUPRC (AUROC) of XGBoost and SVMs (AdaBoost). The effectiveness of sampling in improving the AUROC of the LDA agreed with the findings of a previous study [36].

Regarding the performance reduction by sampling, regularized logistic regression was less affected than the others. In terms of AUPRC, we found that lasso, ridge, and elastic net were more resistant to performance degradation due to sampling than the others, on average. The reduction in AUROC was less pronounced for the linear classifiers, i.e., the LDA and regularized logistic regression methods, compared with the non-linear ones. SVMs were by far the most negatively affected by sampling in AUROC. Indeed, SVMs have been reported to underperform when coupled with undersampling [12].

### Performance comparison of combinations of machine learning and sampling methods

In the previous subsections, we focused on whether sampling improved or reduced the classification performance of a particular machine learning algorithm. Although sampling enhances the performance of machine learning methods, the method could perform worse than other machine learning methods without sampling. To investigate whether sampling was helpful for achieving the best performance for imbalanced classification, we compared the classification performance of each combination of machine learning and sampling methods, including machine learning without sampling. Tables 1 and 2 summarize the comparison results by showing the number of datasets on which a combination achieved the best classification performance in terms of the AUPRC and AUROC, respectively. Note that more than one combination could perform best on a dataset. Furthermore, a combination was regarded to be the best if its performance was not significantly different from that of the best one by the two-tailed 5x2 CV paired *t*-test (*P* ≥ 0.05).

**Table 1:** Number of datasets on which a combination of machine learning and sampling methods performed the best in terms of the area under the precision-recall curve.

	Ada Boost	XG Boost	RF	SVM	LDA	Lasso	Ridge	Elastic net	All
Without sampling	14	21	23	17	13	10	10	11	29
O_Random	11	22	23	9	13	11	11	11	29
O_SMOTE	13	20	23	13	15	11	13	11	29
O_Border	10	16	22	11	12	9	11	9	28
U_Random	9	11	16	10	11	11	10	10	23
U_Condensed	9	14	19	12	10	8	9	9	22
U_NearMiss	5	11	9	8	5	6	6	6	13
SMOTETomek	14	20	22	13	15	11	13	11	28
All	18	25	26	19	16	13	15	14	

Seven sampling methods were compared: random oversampling (O_Random), synthetic minority oversampling technique (O_SMOTE), borderline synthetic minority oversampling technique (O_Border), random undersampling (U_Random), condensed nearest neighbors undersampling (U_Condensed), NearMiss2 (U_NearMiss), and SMOTETomek. Eight machine learning methods were compared: adaptive boosting (AdaBoost), extreme gradient boosting (XGBoost), random forests (RFs), support vector machines (SVMs), linear discriminant analysis (LDA), lasso, ridge, and elastic net. ‘All’ means the number considering all sampling methods (including without sampling) or machine learning methods.

**Table 2:** Number of datasets on which a combination of machine learning and sampling methods performed the best in terms of the area under the receiver operating characteristics curve.

	Ada Boost	XG Boost	RF	SVM	LDA	Lasso	Ridge	Elastic net	All
Without sampling	14	19	24	17	13	14	16	14	30
O_Random	13	19	21	8	13	17	18	17	29
O_SMOTE	15	18	25	11	13	17	18	17	30
O_Border	11	15	21	9	13	13	15	14	28
U_Random	10	16	20	11	14	16	15	16	27
U_Condensed	7	13	17	12	10	13	13	12	23
U_NearMiss	7	9	8	7	7	7	7	7	14
SMOTETomek	16	18	25	12	13	17	18	17	29
All	19	26	28	23	18	17	18	17	

Seven sampling methods were compared: random oversampling (O_Random), synthetic minority oversampling technique (O_SMOTE), borderline synthetic minority oversampling technique (O_Border), random undersampling (U_Random), condensed nearest neighbors undersampling (U_Condensed), NearMiss2 (U_NearMiss), and SMOTETomek. Eight machine learning methods were compared: adaptive boosting (AdaBoost), extreme gradient boosting (XGBoost), random forests (RFs), support vector machines (SVMs), linear discriminant analysis (LDA), lasso, ridge, and elastic net. ‘All’ means the number considering all sampling methods (including without sampling) or machine learning methods.

For most datasets, sampling was not essential for attaining the best classification performance in terms of the AUPRC (29 of 31 datasets) and AUROC (30 of 31 datasets). The appropriate choice of machine learning algorithms without sampling was enough to obtain the optimal result for those datasets. Moreover, no sampling method showed the best performance on more datasets than “without sampling.” O_Random and O_SMOTE achieved the highest AUPRC values on the same number (29) of datasets as “without sampling.” In terms of AUROC, only O_SMOTE performed best on the same number (30) of datasets as “without sampling.” Other sampling methods achieved the best performance on fewer datasets, suggesting that many sampling methods could be more harmful than beneficial for imbalanced classification. For building the optimal classifier, undersampling was worse than oversampling and hybrid methods, as shown in Tables 1 and 2. Regarding these results, we did not observe a considerable difference between the categories of datasets (see Materials and Methods). Sampling was not required for achieving the best performance on most originally binary and multiclass datasets (see S6–S9 Tables).

### Two examples showing contradictory evaluation results depending on the performance measure

In the previous subsections, we found that the evaluation results of the effectiveness of sampling differed according to the performance measures. More precisely, the positive (negative) effect of sampling for imbalanced classification was more (less) pronounced when we measured the performance in terms of AUROC than in terms of AUPRC (see Figs 2 and 3). Because a classifier with the optimal AUROC is not guaranteed to attain the optimal AUPRC [48], using the inappropriate performance measure could mislead the decision-making process.

In this regard, we show two remarkable examples in which the direction of performance changed, i.e., improvement or reduction was reversed depending on the performance measure. The AUROC of the LDA on the Letter_a and the Fraud_Detection datasets (see S1 Table) was significantly improved (paired *t*-tests *P* < 0.05) due to four sampling methods, i.e., O_Random, O_SMOTE, U_Random, and SMOTETomek. In comparison, the AUPRC of the same classification method on the same dataset was significantly reduced (paired *t*-tests *P* < 0.05) by the same four sampling methods. Tables 3 and 4 respectively show the performance of the LDA with and without the four sampling methods on the Letter_a and the Fraud_Detection datasets.

**Table 3:** Performance of linear discriminant analysis (LDA) on the Letter_a dataset with and without the four sampling methods.

Sampling methods	AUPRC		AUROC	
	Mean±standard deviation	*P* values	Mean±standard deviation	*P* values
Without sampling	0.8917±0.0097	N/A	0.9765±0.0041	N/A
O_Random	0.8457±0.0217	(D) 0.0169	0.9851±0.0038	(I) 0.0017
O_SMOTE	0.8412±0.0209	(D) 0.0123	0.9848±0.0038	(I) 0.0016
U_Random	0.8506±0.0217	(D) 0.0050	0.9851±0.0037	(I) 0.0012
SMOTETomek	0.8412±0.0209	(D) 0.0123	0.9848±0.0038	(I) 0.0016

Four sampling methods were compared: random oversampling (O_Random), synthetic minority oversampling technique (O_SMOTE), random undersampling (U_Random), and SMOTETomek. Performances were evaluated by the areas under the precision-recall curve (AUPRC) and the receiver operating characteristics curve (AUROC). The means and standard deviations of 5x2 cross-validation (CV) are shown. *P* values were calculated by the one-tailed 5x2 CV paired *t*-test. (D) and (I) in front of the *P* values represent “decreased” and “increased” compared to the performance of the LDA without sampling, respectively.

**Table 4:** Performance of linear discriminant analysis (LDA) on the Fraud_Detection dataset with and without the four sampling methods.

Sampling methods	AUPRC		AUROC	
	Mean±standard deviation	*P* values	Mean±standard deviation	*P* values
Without sampling	0.4474±0.0038	N/A	0.8697±0.0014	N/A
O_Random	0.3978±0.0056	(D) 0.0029	0.8813±0.0010	(I) 0.0015
O_SMOTE	0.4005±0.0043	(D) 0.0056	0.8802±0.0013	(I) 0.0031
U_Random	0.3547±0.0086	(D) 0.0003	0.8796±0.0010	(I) 0.0031
SMOTETomek	0.4005±0.0043	(D) 0.0056	0.8802±0.0013	(I) 0.0031

Four sampling methods are compared: random oversampling (O_Random), synthetic minority oversampling technique (O_SMOTE), random undersampling (U_Random), and SMOTETomek. Performances were evaluated by the areas under the precision-recall curve (AUPRC) and the receiver operating characteristics curve (AUROC). The means and standard deviations of 5x2 cross-validation (CV) are shown. *P* values were calculated by the one-tailed 5x2 CV paired *t*-test. (D) and (I) in front of the *P* values represent “decreased” and “increased” compared to the performance of the LDA without sampling, respectively.

**Fig 6:**
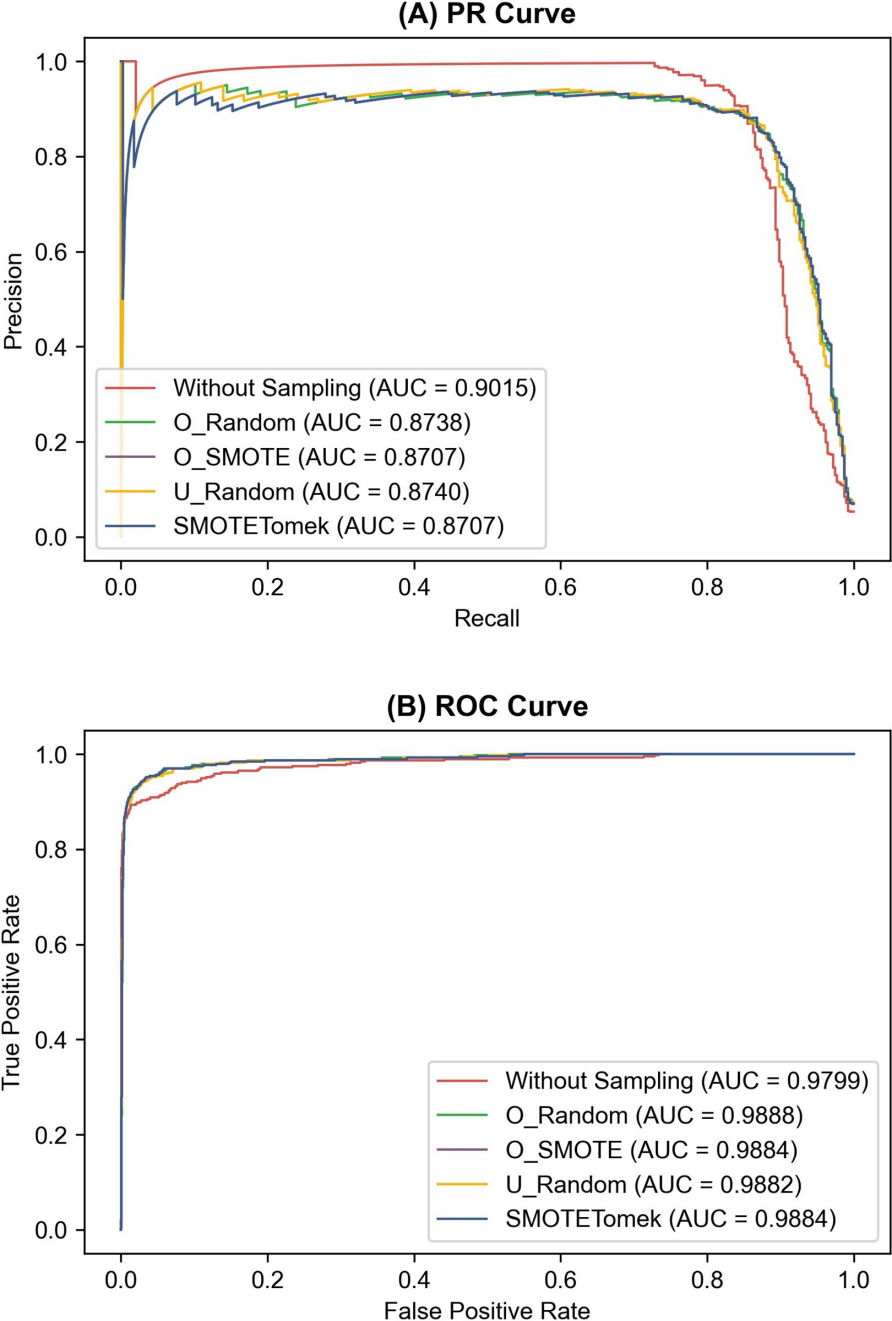
(A) Precision-recall (PR) and (B) receiver operating characteristics (ROC) curves of linear discriminant analysis with and without the four sampling methods on the Letter_a dataset. The PR and ROC curves on the test dataset of the first fold of the first iteration of the 5x2 cross-validation run are shown. Four sampling methods were compared: random oversampling (O_Random), synthetic minority oversampling technique (O_SMOTE), random undersampling (U_Random), and SMOTETomek. AUC indicates the area under the PR or ROC curve.

**Fig 7:**
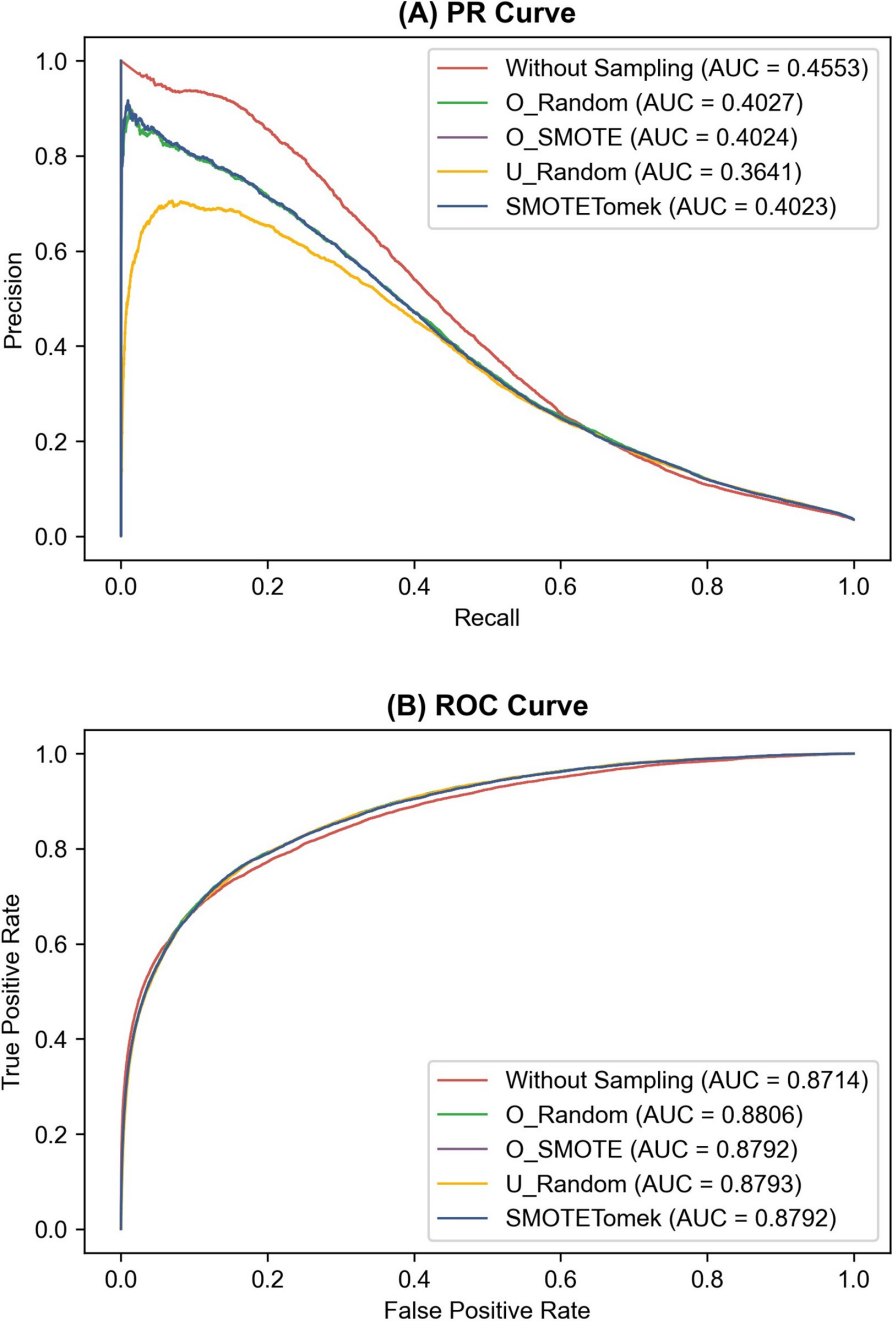
(A) Precision-recall (PR) and (B) receiver operating characteristics (ROC) curves of linear discriminant analysis with and without the four sampling methods on the Fraud_Detection dataset. The PR and ROC curves on the test dataset of the first fold of the first iteration of the 5x2 cross-validation run are shown. Four sampling methods were compared: random oversampling (O_Random), synthetic minority oversampling technique (O_SMOTE), random undersampling (U_Random), and SMOTETomek. AUC means the area under the PR or ROC curve.

The LDA without sampling achieved a higher AUPRC value (0.8917±0.0097) than the best sampling method, i.e., U_Random (0.8506±0.0217), on the Letter_a dataset, which is a binary dataset obtained from a multiclass dataset (see Materials and Methods and S2 Table). In contrast, the AUROC value without sampling (0.9765±0.0041) was lower than those obtained using O_SMOTE and SMOTETomek (0.9848±0.0038), which was the lowest of the four sampling methods on the same dataset. We observed the same results on the Fraud_Detection dataset, which was originally binary (see S2 Table). The LDA without sampling achieved a higher AUPRC value (0.4474±0.0038) than O_SMOTE and SMOTETomek, which performed the best on the Fraud_Detection dataset in terms of AUPRC (0.4005±0.043). On the same dataset, the AUROC value without sampling (0.8697±0.0014) was lower than U_Random (0.8796±0.0010), which was the lowest among those for the four sampling methods. To understand this difference intuitively, we compared the precision-recall (PR) and receiver operating characteristics (ROC) curves on the test dataset of the first fold of the first iteration of the 5x2 CV run (see Materials and Methods and Fig 1) on the two datasets. We show the two curves respectively in Figs 6 and 7.

Figs 6 and 7 show that the sampling dramatically decreases the precision, especially when the recall is close to zero, leading to decreased AUPRC values. In comparison, the AUROC is improved by sampling because the false positive rate decreases more slowly by sampling as the true positive rate (i.e., recall) approaches one. We observed similar trends from the other folds and iterations of the 5x2 CV run (see S2 and S3 Figs).

## Conclusions and discussion

Imbalanced classification is a critical issue in machine learning that is observed in numerous application areas. Sampling methods are among the most popular approaches to solve imbalanced classification problems owing to their ease of use and broad applicability. Although data balancing by sampling could help to build a classifier that is not biased toward the majority class, it distorts the distribution of training data, potentially reducing the test performance. However, their effectiveness has not been comprehensively tested using a wide array of machine learning algorithms.

In this study, we evaluated seven sampling methods for imbalanced classification combined with eight machine learning methods on 31 real-world imbalanced datasets. We observed that sampling affected only a small number of cases. Notably, sampling was more likely to deteriorate than improve the performance of a classifier. This result seems to disagree with those of previous studies [12, 24, 25, 29–38] which suggested that sampling is a remedy for the imbalanced classification problem. Our results were largely in accordance with the results of these studies. Many of the previous studies [24, 25, 29–33] used decision trees which were not covered in our study. Thus, sampling could be a good option when using decision trees for imbalanced classification. Some previous studies used the machine learning methods covered in our study such as LDA [35, 36], RFs [37, 38], and SVMs with the radial basis function kernel [12]. The previous results on LDA [14, 35, 36] partly agree with ours as we also observed the relatively positive effect of sampling on LDA, especially when the classification performance was measured by AUROC (see Fig 5B). The results of the other previous studies were not consistent with ours possibly due to the small number of datasets used in the experiments [12, 38] or some uncharacterized differences in the experimental setting [37].

Among the sampling methods, O_Random and O_SMOTE performed better than the others to improve classification performance. Undersampling reduced the performance more often than others. Thus, we conclude that oversampling is generally better than undersampling in terms of the classification performance. Another important aspect to consider when adopting a sampling method is its efficiency. The analysis of time complexity of each of the seven sampling methods is detailed in S1 File. Among the sampling methods, the most efficient one was U_Random, the every-case time complexity of which is *T*(*n*_*minor*_), where *n*_*minor*_ denotes the number of minority class examples, which is usually much smaller than the training dataset size. U_Condensed and SMOTETomek are the least efficient. The every-case time complexity of U_Condensed is *T*((*n*_*major*_− 1)((*n*– 1)*d* + (*n*– 1))), where *n*_*major*_ denotes the number of majority class examples, *n* = *n*_*major*_ + *n*_*minor*_, and *d* is the number of features. U_Condensed would be especially inefficient when applied to a severely imbalanced dataset, where *n*_*major*_ is similar to *n*. The every-case time complexity of SMOTETomek is *T*((*n*_*major*_−*n*_*minor*_)((*n*_*minor*_− 1)*d* + *k*(*n*_*minor*_− 1))*d* + 2*n*_*major*_((2*n*_*major*_− 1)*d* + (2*n*_*major*_− 1))), where *k* denotes the number of nearest neighbors used for synthesizing a new example (see S1 File). Among the two best performing sampling methods, i.e., O_Random and O_SMOTE, O_Random performed better in terms of time complexity: *T*((*n*_*major*_−*n*_*minor*_)*d*) vs *T*((*n*_*major*_−*n*_*minor*_)((*n*_*minor*_− 1)*d* + *k*(*n*_*minor*_− 1))*d*). Thus, we propose using O_Random if a sampling method is required.

Compared with non-linear classifiers, the linear ones were more likely to be enhanced by sampling. The choice of performance measure had a crucial impact on the evaluation of the sampling methods. The adverse effect of sampling was more pronounced when the performance was measured using the AUPRC than AUROC. In this regard, we found two interesting examples in which the validation results were reversed depending on the performance measure used. This finding is important because AUPRC is known to be preferable to AUROC when measuring the performance of classifiers on imbalanced datasets [39–41]. It has not been observed from the previous studies. To the best of our knowledge, we are the first to comprehensively evaluate sampling methods using the AUPRC.

Our study provides useful insights on the effectiveness of data balancing by sampling for imbalanced classification. We found that sampling could be ineffective or harmful and is not essential to achieve the optimal classifier from an experiment on a large number of imbalanced classification datasets. These findings have not been identified from the previous studies because of differences in the machine learning method evaluated and the limited number of datasets. Based on our findings, we propose to validate the effectiveness of the sampling methods using multiple machine learning algorithms and an appropriate performance measure before using it. Instead of sampling, one could also use the algorithm level and cost-sensitive learning-based approaches to alleviate the problems caused by imbalanced class ratio, although developing these techniques is challenging. Several such approaches [19–22] have been proposed that are applicable to various imbalanced classification problems.

Directions for future work include evaluating the effectiveness of sampling on multiclass imbalanced datasets and comparing the sampling methods with other approaches such as the algorithm level and the cost-sensitive learning-based approaches. Multiclass imbalanced problems are known to be more difficult than their binary counterparts [51] because more factors, e.g., the configurations with classes of intermediate sizes, have a considerable impact on the classification result. A set of sampling approaches to multiclass imbalanced classification have been proposed [52, 53]. It would be helpful to comprehensively evaluate these approaches using a large number of multiclass imbalanced datasets. Recently, a set of algorithm level approaches, e.g., density-weighted support vector machines [19], the intuitionistic fuzzy kernel ridge regression classifier [20], and kernel-target alignment based fuzzy Lagrangian twin bounded support vector machines [22], and a cost-sensitive learning-based approach, i.e., the robust twin bounded support vector machine [21], were proposed for imbalanced classification problems. Because these methods were shown to perform well on many imbalanced datasets, it would be interesting to compare them with sampling approaches.

## Supporting information

S1 Dataset Hyperparameter values of the machine learning methods optimized with respect to the area under the precision-recall curve. (XLSX)

S2 Dataset Hyperparameter values of the machine learning methods optimized with respect to the area under the receiver operating characteristics curve. (XLSX)

S1 Table Descriptions of the 31 datasets used in the experiments. (DOCX)

S2 Table Descriptions of the minority class of the 31 datasets. (DOCX)

S3 Table URL for the 31 datasets. (DOCX)

S4 Table Ratios of the majority to minority classes (in fraction form) of the training dataset modified using the condensed nearest neighbors undersampling method. (DOCX)

S5 Table Optimized hyperparameters of the eight machine learning methods. (DOCX)

S6 Table Number of originally binary datasets on which a combination of machine learning and sampling methods performed the best in terms of the area under the precision-recall curve. (DOCX)

S7 Table Number of originally multiclass datasets on which a combination of machine learning and sampling methods performed the best in terms of the area under the precision-recall curve. (DOCX)

S8 Table Number of originally binary datasets on which a combination of machine learning and sampling methods performed the best in terms of the area under the receiver operating characteristics curve. (DOCX)

S9 Table Number of originally multiclass datasets on which a combination of machine learning and sampling methods performed the best in terms of the area under the receiver operating characteristics curve. (DOCX)

S1 Fig Relationship between the imbalance ratio and the number of cases of performance changes for the 31 datasets. (DOCX)

S2 Fig (A), (C), (E), (G), (I), (K), (M), (O), and (Q) Precision-recall (PR) and (B), (D), (F), (H), (J), (L), (N), (P), and (R) receiver operating characteristics (ROC) curves of linear discriminant analysis with and without four sampling methods on the Letter_a dataset.
(DOCX)

S3 Fig (A), (C), (E), (G), (I), (K), (M), (O), and (Q) Precision-recall (PR) and (B), (D), (F), (H), (J), (L), (N), (P), and (R) receiver operating characteristics (ROC) curves of linear discriminant analysis with and without four sampling methods on the Fraud_Detection dataset.
(DOCX)

S1 File (DOCX)
