# Supplementary material for: An empirical evaluation of sampling methods for the classification of imbalanced data
Source: PLoS One. 2022 Jul 28;17(7):e0271260. doi: 10.1371/journal.pone.0271260 (PMC9333262; doi:10.1371/journal.pone.0271260)
Supplement: S1 Table — (DOCX) [file pone.0271260.s003.docx]

**S1 Table. Descriptions of the 31 datasets used in the experiments.**

Kaggle (<https://www.kaggle.com>) is a public data platform. UCI means the UC Irvine Machine Learning Repository (<https://archive.ics.uci.edu>). The imbalance ratio is defined as (#Majority class examples / #Minority class examples).

| Source | Name | #Examples | #Minority class examples | #Majority class examples | #Features | Imbalance ratio |
| --- | --- | --- | --- | --- | --- | --- |
| Kaggle | Creditcard | 284,807 | 492 | 284,315 | 30 | 577.88 |
| UCI | Shuttle3 | 58,000 | 171 | 57,829 | 9 | 338.18 |
| UCI | Covtype4 | 581,012 | 2,747 | 578,265 | 54 | 210.51 |
| UCI | Abalone19 | 4,177 | 32 | 4,145 | 10 | 129.53 |
| UCI | Abalone_over20 | 4,177 | 62 | 4,115 | 10 | 66.37 |
| UCI | Yeast6 | 1,484 | 35 | 1,449 | 8 | 41.40 |
| UCI | Yeast5 | 1,484 | 44 | 1,440 | 8 | 32.73 |
| UCI | Yeast4 | 1,484 | 51 | 1,433 | 8 | 28.10 |
| Kaggle | Fraud_Detection | 590,540 | 20,663 | 569,877 | 410 | 27.57 |
| UCI | Letter_a | 20,000 | 789 | 19,211 | 16 | 24.35 |
| UCI | Abalone9vs18 | 731 | 42 | 689 | 10 | 16.40 |
| UCI | Glass5 | 214 | 13 | 201 | 9 | 15.46 |
| UCI | Balance_B | 625 | 49 | 576 | 4 | 11.76 |
| UCI | Pendigit9 | 10,992 | 1,055 | 9,937 | 16 | 9.42 |
| UCI | Pageblocks1 | 5,472 | 559 | 4,913 | 10 | 8.79 |
| UCI | Ecoli_imU | 336 | 35 | 301 | 7 | 8.60 |
| UCI | Segment_G | 2,310 | 330 | 1,980 | 18 | 6.00 |
| UCI | Ecoli_pp | 336 | 52 | 284 | 7 | 5.46 |
| UCI | Ecoli_im | 336 | 77 | 259 | 7 | 3.36 |
| UCI | Vehicle_VAN | 846 | 199 | 647 | 18 | 3.25 |
| UCI | Parkinsons_H | 195 | 48 | 147 | 23 | 3.06 |
| UCI | Vehicle_Bus | 846 | 218 | 628 | 18 | 2.88 |
| UCI | Haberman_Died | 306 | 81 | 225 | 3 | 2.78 |
| UCI | Wine3 | 178 | 48 | 130 | 13 | 2.71 |
| UCI | German_Bad | 1,000 | 300 | 700 | 24 | 2.33 |
| UCI | Glass1 | 214 | 70 | 144 | 9 | 2.06 |
| UCI | Iris_Setosa | 150 | 50 | 100 | 4 | 2.00 |
| UCI | Ionosphere_Bad | 351 | 126 | 225 | 34 | 1.79 |
| UCI | Spambase0 | 4,601 | 1,813 | 2,788 | 57 | 1.54 |
| UCI | Heart_H | 270 | 120 | 150 | 13 | 1.25 |
| UCI | Sonar_R | 208 | 97 | 111 | 60 | 1.14 |
