# Supplementary material for: An empirical evaluation of sampling methods for the classification of imbalanced data
Source: PLoS One. 2022 Jul 28;17(7):e0271260. doi: 10.1371/journal.pone.0271260 (PMC9333262; doi:10.1371/journal.pone.0271260)
Supplement: S2 Table — (DOCX) [file pone.0271260.s004.docx]

**S2 Table. Descriptions of the minority class of the 31 datasets.**

| Datasets | Original class type | Classes in the original dataset | Minority class |
| --- | --- | --- | --- |
| Creditcard | Binary | 0, 1 | 1 |
| Shuttle3 | Multi | 1, 2, 3, 4, 5, 6, 7 | 3 |
| Covtype4 | Multi | 1, 2, 3, 4, 5, 6, 7 | 4 |
| Abalone19 | Multi | 1 to 29 (integer) | 19 |
| Abalone_over20 | Multi | 1 to 29 (integer) | > 20 |
| Yeast6 | Multi | ‘CYT’, ‘NUC’, ‘MIT’, ‘ME3’, ‘ME2’, ‘ME1’, ‘EXC’, ‘VAC’, ‘PRX’, ‘ERL’ | ‘EXC’ |
| Yeast5 | Multi | ‘CYT’, ‘NUC’, ‘MIT’, ‘ME3’, ‘ME2’, ‘ME1’, ‘EXC’, ‘VAC’, ‘PRX’, ‘ERL’ | ‘ME1’ |
| Yeast4 | Multi | ‘CYT’, ‘NUC’, ‘MIT’, ‘ME3’, ‘ME2’, ‘ME1’, ‘EXC’, ‘VAC’, ‘PRX’, ‘ERL’ | ‘ME2’ |
| Fraud_Detection | Binary | 0, 1 | 1 |
| Letter_a | Multi | ‘A’ to ‘Z’ (alphabet capital letters) | ‘A’ |
| Abalone9vs18 | Multi | 9, 18 | 9 |
| Glass5 | Multi | 1, 2, 3, 4, 5, 6, 7 | 5 |
| Balance_B | Multi | ‘L’, ‘B’, ‘R’ | ‘B’ |
| Pendigit9 | Multi | 0, 1, 2, 3, 4, 5, 6, 7, 8, 9 | 9 |
| Pageblocks1 | Multi | 1, 2, 3, 4, 5 | 1 |
| Ecoli_imU | Multi | ‘cp’, ‘im’, ‘imL’, ‘imS’, ‘imU’, ‘om’, ‘omL’, ‘pp’ | ‘imU’ |
| Segment_G | Multi | ‘BRICKFACE’, ‘CEMENT’, ‘FOLIAGE’, ‘GLASS’, ‘PATH’, ‘SKY’, ‘WINDOW’ | ‘GRASS’ |
| Ecoli_pp | Multi | ‘cp’, ‘im’, ‘imL’, ‘imS’, ‘imU’, ‘om’, ‘omL’, ‘pp’ | ‘pp’ |
| Ecoli_im | Multi | ‘cp’, ‘im’, ‘imL’, ‘imS’, ‘imU’, ‘om’, ‘omL’, ‘pp’ | ‘im’ |
| Vehicle_VAN | Multi | ‘OPEL’, ‘SAAB’, ‘BUS’, ‘VAN’ | ‘VAN’ |
| Parkinsons_H | Binary | 0, 1 | 0 |
| Vehicle_Bus | Multi | ‘OPEL’, ‘SAAB’, ‘BUS’, ‘VAN’ | ‘BUS’ |
| Haberman_Died | Binary | 1, 2 | 2 |
| Wine3 | Multi | 1, 2, 3 | 3 |
| German_Bad | Binary | ‘Good’, ‘Bad’ | ‘Bad’ |
| Glass1 | Multi | 1, 2, 3, 4, 5, 6, 7 | 1 |
| Iris_Setosa | Multi | ‘Setosa’, ‘Versicolour’, ‘Virginica’ | ‘Setosa’ |
| Ionosphere_Bad | Binary | ‘Good’, ‘Bad’ | ‘Bad’ |
| Spambase0 | Binary | 0, 1 | 0 |
| Heart_H | Binary | ‘H’, ‘A’ | ‘H’ |
| Sonar_R | Binary | ‘R’, ‘M’ | ‘R’ |
