# Supplementary material for: An empirical evaluation of sampling methods for the classification of imbalanced data
Source: PLoS One. 2022 Jul 28;17(7):e0271260. doi: 10.1371/journal.pone.0271260 (PMC9333262; doi:10.1371/journal.pone.0271260)
Supplement: S3 Table — (DOCX) [file pone.0271260.s005.docx]

**S3 Table. URL for the 31 datasets.**

| Datasets | Name in the source repository | URL |
| --- | --- | --- |
| Creditcard | Creditcard | https://www.kaggle.com/datasets/mlg-ulb/creditcardfraud |
| Shuttle3 | Statlog (Shuttle) | https://archive.ics.uci.edu/ml/datasets/Statlog+(Shuttle) |
| Covtype4 | Covertype | https://archive.ics.uci.edu/ml/datasets/covertype |
| Abalone19 | Abalone | https://archive.ics.uci.edu/ml/datasets/abalone |
| Abalone_over20 | Abalone | https://archive.ics.uci.edu/ml/datasets/abalone |
| Yeast6 | Yeast | https://archive.ics.uci.edu/ml/datasets/Yeast |
| Yeast5 | Yeast | https://archive.ics.uci.edu/ml/datasets/Yeast |
| Yeast4 | Yeast | https://archive.ics.uci.edu/ml/datasets/Yeast |
| Fraud_Detection | Fraud Detection | https://www.kaggle.com/competitions/ieee-fraud-detection/data |
| Letter_a | Letter Recognition | https://archive.ics.uci.edu/ml/datasets/letter+recognition |
| Abalone9vs18 | Abalone | https://archive.ics.uci.edu/ml/datasets/abalone |
| Glass5 | Glass Identification | https://archive.ics.uci.edu/ml/datasets/glass+identification |
| Balance_B | Balance Scale | https://archive.ics.uci.edu/ml/datasets/balance+scale |
| Pendigit9 | Pen-Based Recognition of Handwritten Digits | https://archive.ics.uci.edu/ml/datasets/pen-based+recognition+of+handwritten+digits |
| Pageblocks1 | PageBlocks Classification | https://archive.ics.uci.edu/ml/datasets/Page+Blocks+Classification |
| Ecoli_imU | Ecoli | https://archive.ics.uci.edu/ml/datasets/ecoli |
| Segment_G | Statlog (ImageSegmentation) | https://archive.ics.uci.edu/ml/datasets/Statlog+(Image+Segmentation) |
| Ecoli_pp | Ecoli | https://archive.ics.uci.edu/ml/datasets/ecoli |
| Ecoli_im | Ecoli | https://archive.ics.uci.edu/ml/datasets/ecoli |
| Vehicle_VAN | Statlog (Vehicle Silhouettes) | https://archive.ics.uci.edu/ml/datasets/Statlog+%28Vehicle+Silhouettes%29 |
| Parkinsons_H | Parkinsons | https://archive.ics.uci.edu/ml/datasets/parkinsons |
| Vehicle_Bus | Statlog (Vehicle Silhouettes) | https://archive.ics.uci.edu/ml/datasets/Statlog+%28Vehicle+Silhouettes%29 |
| Haberman_Died | Haberman's Survival | https://archive.ics.uci.edu/ml/datasets/haberman%27s+survival |
| Wine3 | Wine | https://archive.ics.uci.edu/ml/datasets/wine |
| German_Bad | Statlog  (German Credit Data) | https://archive.ics.uci.edu/ml/datasets/statlog+(german+credit+data) |
| Glass1 | Glass Identification | https://archive.ics.uci.edu/ml/datasets/glass+identification |
| Iris_Setosa | Iris | https://archive.ics.uci.edu/ml/datasets/iris |
| Ionosphere_Bad | Ionosphere | https://archive.ics.uci.edu/ml/datasets/ionosphere |
| Spambase0 | Spambase | https://archive.ics.uci.edu/ml/datasets/spambase |
| Heart_H | Statlog (Heart) | https://archive.ics.uci.edu/ml/datasets/statlog+(heart) |
| Sonar_R | Connectionist Bench (Sonar, Mines vs. Rocks) | https://archive.ics.uci.edu/ml/datasets/connectionist+bench+(sonar,+mines+vs.+rocks) |
