# Supplementary material for: An empirical evaluation of sampling methods for the classification of imbalanced data
Source: PLoS One. 2022 Jul 28;17(7):e0271260. doi: 10.1371/journal.pone.0271260 (PMC9333262; doi:10.1371/journal.pone.0271260)
Supplement: S4 Table — (DOCX) [file pone.0271260.s006.docx]

**S4 Table. Ratios of the majority to minority classes (in fraction form) of the training dataset modified using the condensed nearest neighbors undersampling method.**

The ratio in each fold of each iteration of the 5x2 cross-validation run for the 31 datasets is shown.

| Datasets | Iteration 1 | | Iteration 2 | | Iteration 3 | | Iteration 4 | | Iteration 5 | |
| --- | --- | --- | --- | --- | --- | --- | --- | --- | --- | --- |
|  | Fold 1 | Fold 2 | Fold 1 | Fold 2 | Fold 1 | Fold 2 | Fold 1 | Fold 2 | Fold 1 | Fold 2 |
| Creditcard | 2.9 | 2.6 | 2.8 | 2.9 | 3.0 | 2.7 | 2.2 | 3.3 | 2.5 | 3.1 |
| Shuttle3 | 0.3 | 0.3 | 0.4 | 0.3 | 0.4 | 0.3 | 0.4 | 0.3 | 0.4 | 0.3 |
| Covtype4 | 1.0 | 1.0 | 1.1 | 1.0 | 1.0 | 1.0 | 1.0 | 1.1 | 1.1 | 1.0 |
| Abalone19 | 5.9 | 6.6 | 6.9 | 6.8 | 8.0 | 5.2 | 6.7 | 6.9 | 6.9 | 5.9 |
| Abalone_over20 | 3.8 | 4.5 | 4.4 | 3.4 | 4.3 | 3.7 | 3.8 | 4.0 | 4.3 | 3.9 |
| Yeast6 | 2.5 | 3.4 | 2.8 | 3.2 | 3.9 | 2.0 | 2.8 | 2.9 | 3.9 | 2.1 |
| Yeast5 | 1.8 | 1.9 | 1.5 | 1.8 | 1.9 | 1.5 | 1.7 | 1.6 | 1.6 | 1.8 |
| Yeast4 | 3.4 | 2.8 | 2.6 | 3.5 | 2.9 | 3.1 | 2.6 | 3.3 | 3.5 | 2.9 |
| Fraud_Detection | 2.7 | 2.7 | 2.6 | 2.7 | 2.7 | 2.7 | 2.7 | 2.7 | 2.7 | 2.7 |
| Letter_a | 0.7 | 0.7 | 0.7 | 0.6 | 0.7 | 0.7 | 0.7 | 0.7 | 0.7 | 0.7 |
| Abalone9vs18 | 2.4 | 2.5 | 2.2 | 2.5 | 2.3 | 2.0 | 2.1 | 2.1 | 2.4 | 2.2 |
| Glass5 | 1.3 | 1.3 | 2.0 | 1.1 | 1.8 | 1.1 | 1.3 | 1.3 | 1.8 | 1.1 |
| Balance_B | 3.5 | 3.3 | 3.2 | 3.3 | 2.9 | 3.3 | 3.2 | 3.6 | 3.4 | 3.6 |
| Pendigit9 | 0.1 | 0.2 | 0.2 | 0.2 | 0.2 | 0.2 | 0.1 | 0.2 | 0.2 | 0.2 |
| Pageblocks1 | 0.1 | 0.5 | 0.5 | 0.5 | 0.5 | 0.5 | 0.5 | 0.5 | 0.4 | 0.5 |
| Ecoli_imU | 1.2 | 1.3 | 1.6 | 1.3 | 1.2 | 1.3 | 1.4 | 1.4 | 1.3 | 1.2 |
| Segment_G | 0.1 | 0.1 | 0.1 | 0.1 | 0.1 | 0.1 | 0.1 | 0.1 | 0.1 | 0.1 |
| Ecoli_pp | 1.1 | 1.1 | 1.1 | 0.6 | 0.9 | 0.7 | 1.0 | 1.1 | 1.0 | 0.9 |
| Ecoli_im | 0.7 | 0.6 | 0.7 | 0.7 | 0.6 | 0.7 | 0.8 | 0.6 | 0.7 | 0.6 |
| Vehicle_VAN | 0.6 | 0.5 | 0.5 | 0.5 | 0.5 | 0.6 | 0.5 | 0.6 | 0.6 | 0.6 |
| Parkinsons_H | 0.9 | 0.8 | 0.8 | 0.8 | 1.0 | 0.7 | 0.8 | 0.8 | 0.8 | 0.7 |
| Vehicle_Bus | 0.5 | 0.5 | 0.5 | 0.5 | 0.5 | 0.4 | 0.5 | 0.5 | 0.5 | 0.5 |
| Haberman_Died | 1.5 | 1.4 | 1.4 | 1.2 | 1.5 | 1.3 | 1.5 | 1.4 | 1.2 | 1.4 |
| Wine3 | 0.4 | 0.4 | 0.4 | 0.3 | 0.4 | 0.5 | 0.3 | 0.5 | 0.3 | 0.4 |
| German_Bad | 1.1 | 1.1 | 1.1 | 1.1 | 1.1 | 1.1 | 1.2 | 1.1 | 1.1 | 1.2 |
| Glass1 | 0.7 | 0.9 | 0.8 | 0.8 | 0.8 | 0.9 | 0.8 | 0.8 | 0.7 | 0.9 |
| Iris_Setosa | 0.1 | 0.1 | 0.1 | 0.2 | 0.1 | 0.1 | 0.1 | 0.1 | 0.1 | 0.1 |
| Ionosphere_Bad | 0.3 | 0.2 | 0.3 | 0.2 | 0.3 | 0.3 | 0.2 | 0.3 | 0.3 | 0.2 |
| Spambase0 | 0.4 | 0.4 | 0.4 | 0.4 | 0.4 | 0.4 | 0.4 | 0.3 | 0.4 | 0.4 |
| Heart_H | 0.6 | 0.6 | 0.6 | 0.6 | 0.6 | 0.6 | 0.6 | 0.6 | 0.6 | 0.6 |
| Sonar_R | 0.4 | 0.6 | 0.5 | 0.5 | 0.5 | 0.5 | 0.6 | 0.4 | 0.5 | 0.4 |
