# Supplementary material for: An empirical evaluation of sampling methods for the classification of imbalanced data
Source: PLoS One. 2022 Jul 28;17(7):e0271260. doi: 10.1371/journal.pone.0271260 (PMC9333262; doi:10.1371/journal.pone.0271260)
Supplement: S5 Table — (DOCX) [file pone.0271260.s007.docx]

**S5 Table. Optimized hyperparameters of the eight machine learning methods.**

| Machine learning methods | Hyperparameters and search ranges |
| --- | --- |
| Adaptive boosting | ‘n_estimators’: [10, 50, 100, 500]  ‘learning_rate’: [0.01, 0.1, 1] |
| Extreme gradient boosting | ‘subsample’: [0.6, 0.8, 1.0]  ‘min_child_weight’: [1, 5, 10]  ‘max_depth’: [3, 4, 5, 6]  ‘gamma’: [0, 0.5, 1]  ‘colsample_bytree’: [0.6, 0.8, 1] |
| Random forests | ‘n_estimators’: [100, 500, 1000, 3000] |
| Support vector machines | ‘C’: [0.1, 1, 10]  ‘gamma’: [0.01, 0.1, 1] |
| Linear discriminant analysis | Not applicable |
| Lasso | ‘C’: [0.1, 1, 10] |
| Ridge | ‘C’: [0.1, 1, 10] |
| Elastic net | ‘C’: [0.1, 1, 10] |
