# Supplementary material for: An empirical evaluation of sampling methods for the classification of imbalanced data
Source: PLoS One. 2022 Jul 28;17(7):e0271260. doi: 10.1371/journal.pone.0271260 (PMC9333262; doi:10.1371/journal.pone.0271260)
Supplement: S9 Table — (DOCX) [file pone.0271260.s011.docx]

**S9 Table. Number of originally multiclass datasets on which a combination of machine learning and sampling methods performed the best in terms of the area under the receiver operating characteristics curve.**

Seven sampling methods were compared: random oversampling (O_Random), synthetic minority oversampling technique (O_SMOTE), borderline synthetic minority oversampling technique (O_Border), random undersampling (U_Random), condensed nearest neighbors undersampling (U_Condensed), NearMiss2 (U_NearMiss), and SMOTETomek. Eight machine learning methods were compared: adaptive boosting (AdaBoost), extreme gradient boosting (XGBoost), random forests (RFs), support vector machines (SVMs), linear discriminant analysis (LDA), lasso, ridge, and elastic net. ‘All’ means the number considering all sampling methods (including without sampling) or machine learning methods.

|  | Ada  Boost | XG  Boost | RF | SVM | LDA | Lasso | Ridge | Elastic  net | All |
| --- | --- | --- | --- | --- | --- | --- | --- | --- | --- |
| Without sampling | 12 | 16 | 18 | 13 | 10 | 12 | 13 | 12 | 22 |
| O_Random | 10 | 15 | 16 | 8 | 11 | 13 | 14 | 13 | 21 |
| O_SMOTE | 10 | 13 | 18 | 9 | 11 | 13 | 14 | 13 | 22 |
| O_Border | 8 | 11 | 16 | 9 | 10 | 10 | 12 | 11 | 21 |
| U_Random | 7 | 13 | 15 | 8 | 11 | 12 | 12 | 12 | 20 |
| U_Condensed | 5 | 11 | 14 | 9 | 8 | 11 | 11 | 11 | 19 |
| U_NearMiss | 6 | 7 | 6 | 7 | 5 | 5 | 5 | 5 | 10 |
| SMOTETomek | 11 | 13 | 18 | 9 | 11 | 13 | 14 | 13 | 22 |
| All | 14 | 19 | 20 | 17 | 13 | 13 | 14 | 13 |  |
