# Supplementary material for: An empirical evaluation of sampling methods for the classification of imbalanced data
Source: PLoS One. 2022 Jul 28;17(7):e0271260. doi: 10.1371/journal.pone.0271260 (PMC9333262; doi:10.1371/journal.pone.0271260)
Supplement: S1 Fig — (DOCX) [file pone.0271260.s012.docx]

**
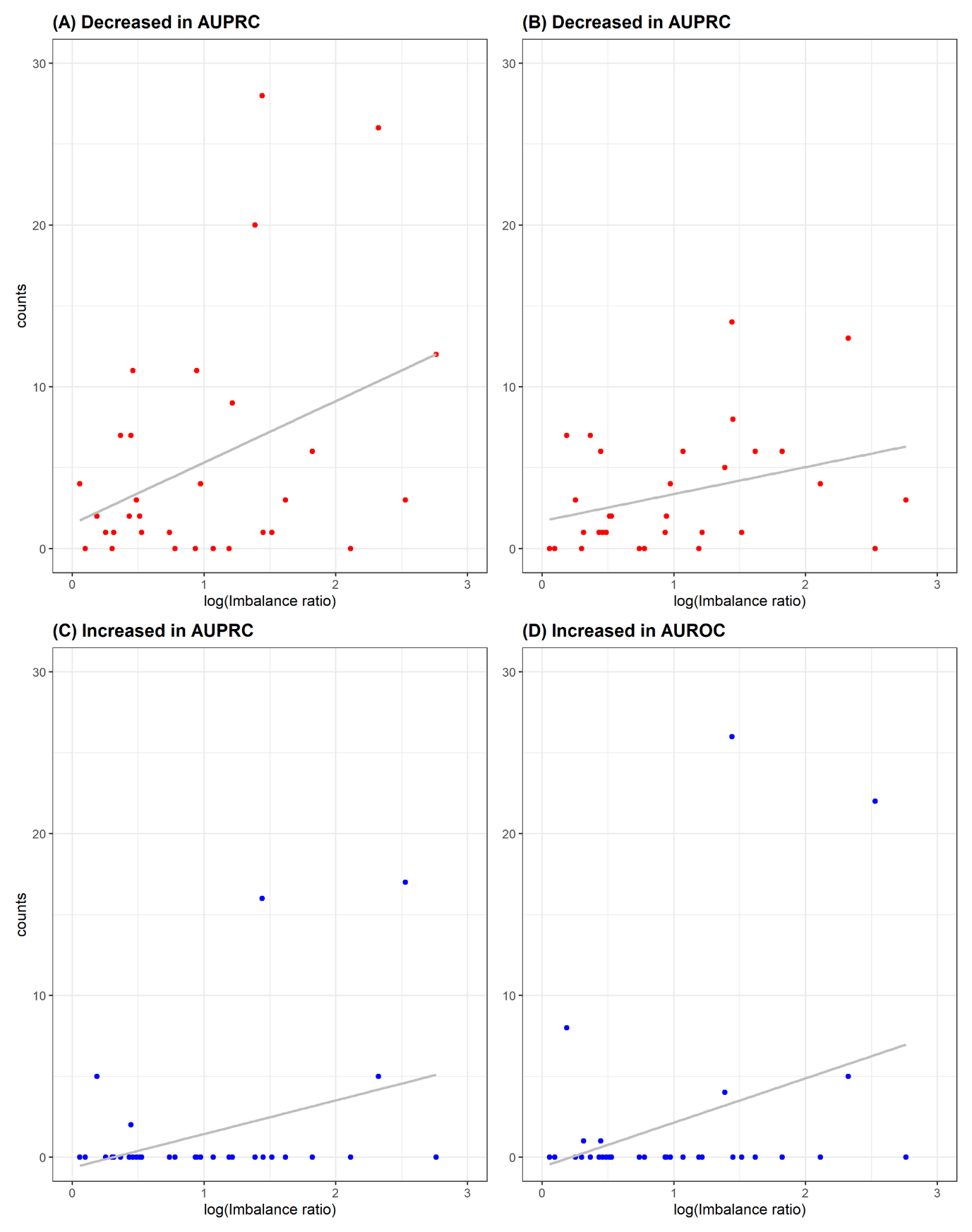
**

**S1 Fig. Relationship between the imbalance ratio and the number of cases of performance changes for the 31 datasets.**

The scatterplots of the imbalance ratio against the number of cases of (A) performance reduction in the area under the precision-recall curve (AUPRC), (B) performance reduction in the area under the receiver operating characteristics curve (AUROC), (C) performance improvement in AUPRC, and (D) performance improvement in AUROC are shown. The imbalance ratio is in a logarithmic scale. Linear regression lines are drawn in the plots.
