# Supplementary material for: An empirical evaluation of sampling methods for the classification of imbalanced data
Source: PLoS One. 2022 Jul 28;17(7):e0271260. doi: 10.1371/journal.pone.0271260 (PMC9333262; doi:10.1371/journal.pone.0271260)
Supplement: S3 Fig — (A), (C), (E), (G), (I), (K), (M), (O), and (Q) Precision-recall (PR) and (B), (D), (F), (H), (J), (L), (N), (P), and (R) receiver operating characteristics (ROC) curves of linear discriminant analysis with and without four sampling methods on the Fraud_Detection dataset. (DOCX) [file pone.0271260.s014.docx]

| Fold and iteration | PR curve | ROC curve |
| --- | --- | --- |
| Fold 2 of iteration 1 | (A)  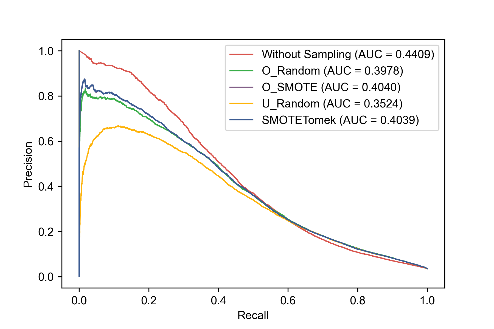 | (B)  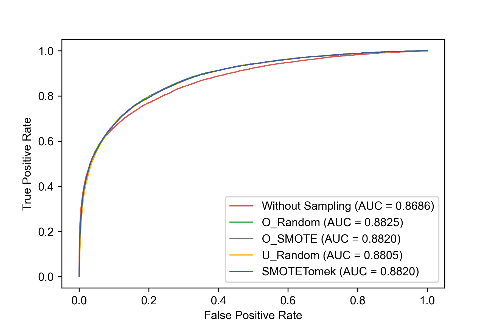 |
| Fold 1 of iteration 2 | (C)  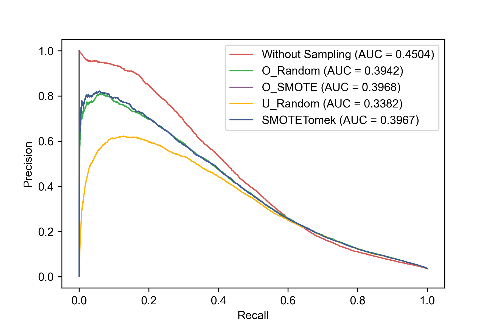 | (D)  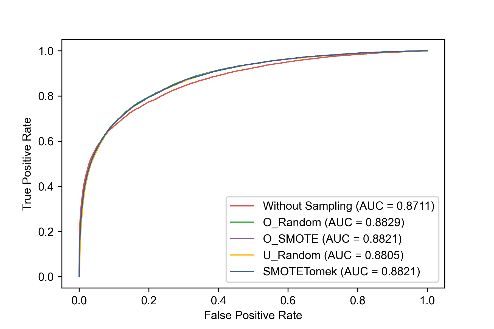 |
| Fold 2 of iteration 2 | (E)  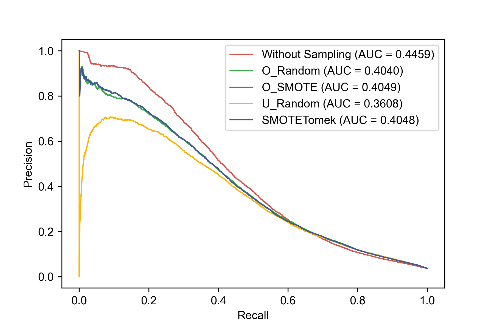 | (F)  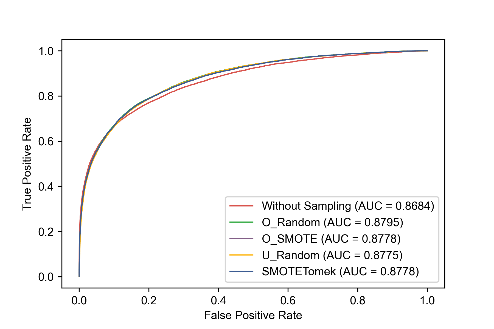 |
| Fold 1 of iteration 3 | (G)  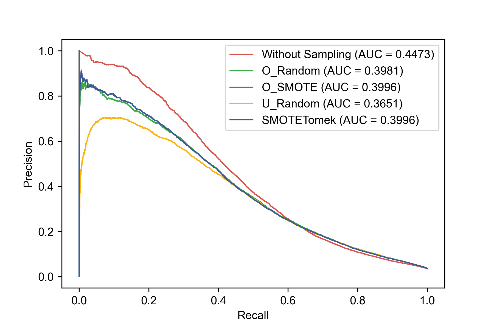 | (H)  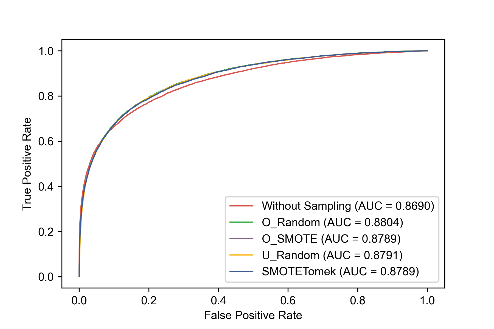 |
| Fold 2 of iteration 3 | (I)  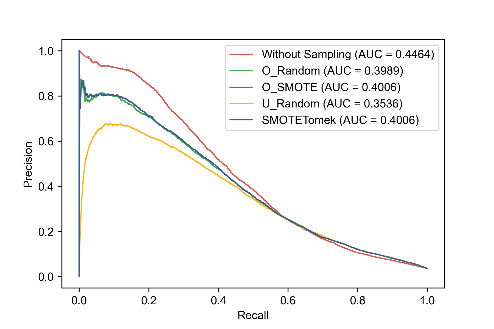 | (J)  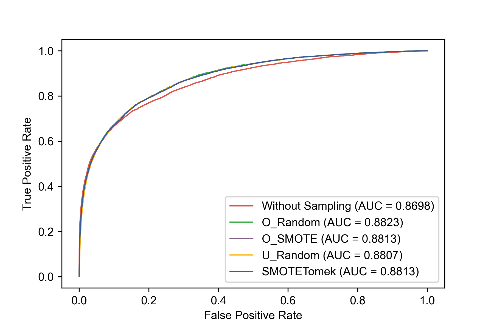 |
| Fold 1 of iteration 4 | (K)  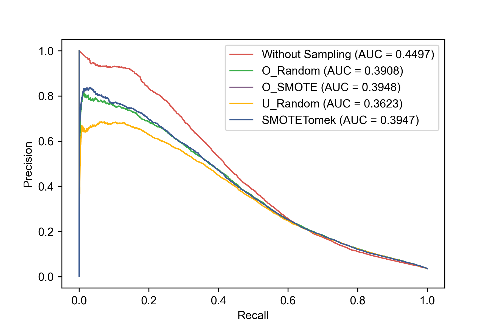 | (L)  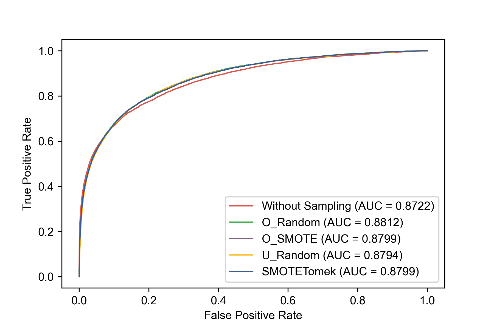 |
| Fold 2 of iteration 4 | (M)  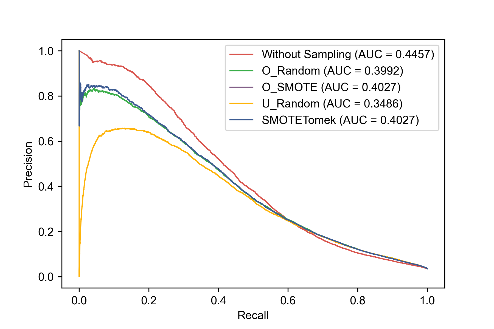 | (N)  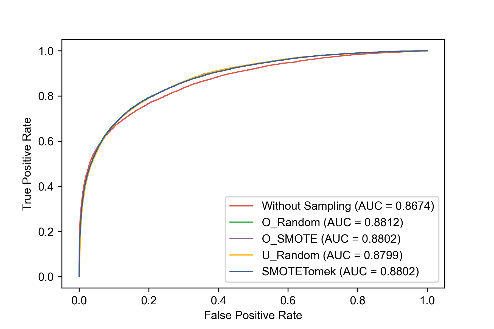 |
| Fold 1 of iteration 5 | (O)  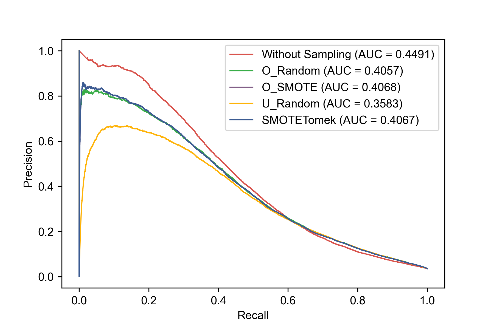 | (P)  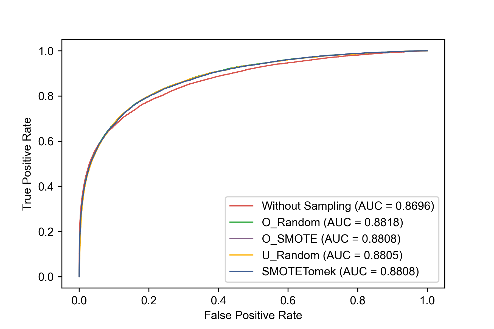 |
| Fold 2 of iteration 5 | (Q)  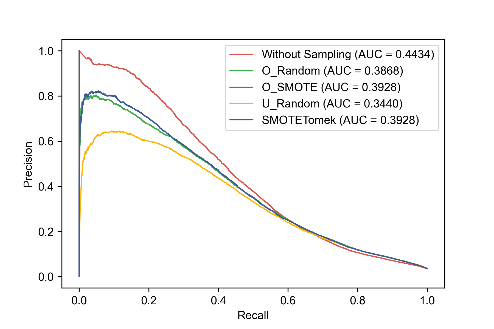 | (R)  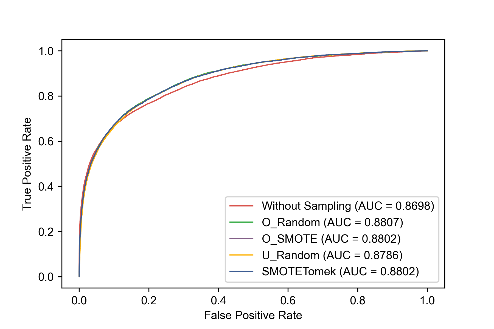 |

S3 Fig. (A), (C), (E), (G), (I), (K), (M), (O), and (Q) Precision-recall (PR) and (B), (D), (F), (H), (J), (L), (N), (P), and (R) receiver operating characteristics (ROC) curves of linear discriminant analysis with and without four sampling methods on the Fraud_Detection dataset.

The PR and ROC curves on the test datasets of the 5x2 cross-validation run are shown. Four sampling methods are random oversampling (O_Random), synthetic minority oversampling technique (O_SMOTE), random undersampling (U_Random), and SMOTETomek. AUC means the area under the PR or ROC curve.
