# Supplementary material for: An empirical evaluation of sampling methods for the classification of imbalanced data
Source: PLoS One. 2022 Jul 28;17(7):e0271260. doi: 10.1371/journal.pone.0271260 (PMC9333262; doi:10.1371/journal.pone.0271260)
Supplement: S1 File — (DOCX) [file pone.0271260.s015.docx]

**Supplementary Methods**

**1. Random oversampling**

Random oversampling is a method of increasing the minority class size by randomly selecting and replicating examples of the minority class. In our experiments, we increased the minority class data so that the ratio of the majority to minority classes was 1:1. To make the class ratio 1:1, we selected and replicated (*n_major_* – *n_minor_*) examples of the minority class, where *n_major_* and *n_minor_* denote the number of the majority and minority class examples in the training dataset, respectively. Thus, the every-case time complexity of random oversampling is *T*((*n_major_* – *n_minor_*)*d*), where *d* denotes the number of features. We called over_sampling.RandomOverSampler from the imbalanced-learn package (version 0.7.0) as follows.

imblearn.over_sampling.RandomOverSampler(ratio='auto', random_state=12345)

**2. Synthetic minority oversampling technique (SMOTE)**

SMOTE [1] synthesizes a new example of the minority class using a randomly selected minority class example and its *k* nearest neighbors of the same class. To generate a new example, the difference in the feature space between the selected example and a randomly selected one of its *k* nearest neighbors of the same class is calculated. The calculated difference is multiplied by a random number between 0 and 1, and the result is added to the selected example, making a new minority class example. In our experiments, *k* was set as 5. The minority class data was increased so that the ratio of the majority to minority classes was 1:1. The every-case time complexity of SMOTE is *T*((*n_major_* – *n_minor_*)((*n_minor_* – 1)*d* + *k*(*n_minor_* – 1))*d*) because the every-case time complexity of finding the *k* nearest minority class examples of a selected minority class example is *T*((*n_minor_* – 1)*d* + *k*(*n_minor_* – 1)). We called over_sampling.SMOTE from the imbalanced-learn package (version 0.7.0) as follows.

imblearn.over_sampling.SMOTE(ratio='auto', random_state=12345, k=None, k_neighbors=5, m=None, m_neighbors=10, out_step=0.5, kind='regular', svm_estimator=None, n_jobs=1)

**3. Borderline SMOTE**

Borderline SMOTE [2] is a variant of SMOTE which focuses on “difficult-to-classify” regions by increasing the minority class data near the borderline. For all the minority class examples, *k* nearest neighbors are selected. A minority class example, more than half of whose *k* nearest neighbors are from the majority class, is labeled “DANGER,” which means that the example is near the borderline and difficult to classify. For the examples labeled “DANGER,” *m* nearest neighbors of the same (i.e., minority) class are selected. A new example is synthesized using a “DANGER” example and its *m* nearest neighbors of the same class as in SMOTE. In our experiments, *k* and *m* were set as 5 and 10, respectively. The minority class data was increased so that the ratio of the majority to minority classes was 1:1. The every-case time complexity for finding “DANGER” examples from the minority class is *T*(*n_minor_*((*n* – 1)*d* + *k*(*n* – 1)), where *n* is *n_major_* + *n_minor_*. Thus, the every-case time complexity of borderline SMOTE is *T*((*n_minor_*((*n* – 1)*d* + *k*(*n* – 1)) + (*n_major_* – *n_minor_*)((*n_minor_* – 1)*d* + *m*(*n_minor_* – 1))*d*). We called over_sampling.BorderlineSMOTE from the imbalanced-learn package (version 0.7.0) as follows.

imblearn.over_sampling.BorderlineSMOTE(ratio='auto', random_state=12345, k=None, k_neighbors=5, m=None, m_neighbors=10, out_step=0.5, kind='regular', svm_estimator=None, n_jobs=1)

**4. Random undersampling**

Random undersampling decreases the majority class data by randomly removing examples of the majority class. In our experiments, we reduced the majority class data so that the ratio of the majority to minority classes was 1:1. Thus, the every-case time complexity of random undersampling is *T*(*n_minor_*). We called under_sampling.RandomUnderSampler from the imbalanced-learn package (version 0.7.0) as follows.

imblearn.under_sampling.RandomUnderSampler(ratio='auto', return_indices=False, random_state=12345, replacement=False)

**5.** **Condensed nearest neighbors undersampling**

Condensed nearest neighbors undersampling [3] is a method of selecting examples of the majority class for training based on their nearest neighbors. We denote the training dataset by **S**. First, **S** contains all the minority class examples. Then, a randomly selected example from the majority class is added to **S**. Finally, the remaining majority class examples are added to **S** if their nearest neighbors are from the minority class. In this manner, **S** contains all the minority class examples and the majority class examples close to the minority class. The every-case time complexity of condensed nearest neighbors undersampling is *T*((*n_major_* – 1)((*n* – 1)*d* + (*n* – 1))). We called under_sampling.CondensedNearestNeighbour from the imbalanced-learn package (version 0.7.0) as follows.

imblearn.under_sampling.CondensedNearestNeighbour(ratio='auto', return_indices=False, random_state=12345, size_ngh=None, n_neighbors=None, n_seeds_S=1, n_jobs=1)

The resulting class distribution varies according to the composition of datasets. The class ratio of the balanced training dataset of each fold of each iteration of the 5x2 cross-validation run (see Materials and Methods) for the 31 datasets is described in S4 Table.

**6. NearMiss2**

NearMiss [4] uses *k* nearest neighbors to select the majority class examples for training. Among the four types of NearMiss, we used the second one, i.e., NearMiss2, which has been demonstrated to perform best for imbalanced classification. In NearMiss2, the average distance of each majority class example from the three farthest minority class examples is calculated. Based on the calculated distances, the majority class examples close to the minority class are chosen for training. In our experiments, we decreased the majority class data so that the ratio of the majority to minority classes was 1:1. The every-case time complexity of NearMiss2 is *T*(*n_major_*(*n_minor_d* + 3*n_minor_*) + *n_major_*log(*n_major_*) + *n_minor_*) if a sorting algorithm, of which every-case time complexity is *T*(*i_size*·log(*i_size*)), where *i_size* denotes the input size, is used. We called under_sampling.NearMiss from the imbalanced-learn package (version 0.7.0) as follows.

imblearn.under_sampling.NearMiss(ratio='auto', return_indices=False, random_state=12345, version=2, size_ngh=None, n_neighbors=3, ver3_samp_ngh=None, n_neighbors_ver3=3, n_jobs=1)

**7. SMOTETomek**

SMOTETomek [5] is a hybrid method in which SMOTE is applied first, and then the Tomek links method (an undersampling method) is performed. A pair of data examples is defined as a Tomek link if they are from different classes and the nearest neighbors of each other [6]. After generating minority data examples using SMOTE, Tomek links are identified, and the majority and minority data examples of the identified Tomek links are removed. In our experiments, we used SMOTE to increase the minority class data so that the ratio of the two classes was 1:1. The Tomek links method does not alter the class distribution because it removes the same number of examples from the majority and minority classes. The every-case time complexity for finding the Tomek links in the training dataset whose size was increased to 2*n_major_* by SMOTE is *T*(2*n_major_*((2*n_major_* – 1)*d* + (2*n_major_* – 1))). Thus, the every-case time complexity of SMOTETomek is *T*((*n_major_* – *n_minor_*)((*n_minor_* – 1)*d* + *k*(*n_minor_* – 1))*d* + 2*n_major_*((2*n_major_* – 1)*d* + (2*n_major_* – 1))). We called combine.SMOTETomek from the imbalanced-learn package (version 0.7.0) as follows.

imblearn.combine.SMOTETomek(ratio='auto', random_state=12345, smote=None, tomek=None, k=None, m=None, out_step=None, kind_smote=None, n_jobs=None)

**References**

1. Chawla NV, Bowyer KW, Hall LO, Kegelmeyer WP. SMOTE: Synthetic minority over-sampling technique. J Artif Intell Res. 2002;16:321-57.

2. Han H, Wang W-Y, Mao B-H, editors. Borderline-SMOTE: A new over-sampling method in imbalanced data sets learning. International Conference on Intelligent Computing; 2005: Springer.

3. Hart P. The condensed nearest neighbor rule (corresp.). IEEE Trans Inf Theory. 1968;14(3):515-6.

4. Mani I, Zhang I, editors. kNN approach to unbalanced data distributions: A case study involving information extraction. Proceedings of workshop on learning from imbalanced datasets; 2003: ICML United States.

5. Batista GE, Prati RC, Monard MC. A study of the behavior of several methods for balancing machine learning training data. SIGKDD Explor. 2004;6(1):20-9.

6. Tomek I. An experiment with the edited nearest-neighbor rule. IEEE Trans Syst Man Cybern. 1976;6(6):448-52.
